# Targeting YAP1/LINC00152/FSCN1 Signaling Axis Prevents the Progression of Colorectal Cancer

**DOI:** 10.1002/advs.201901380

**Published:** 2019-12-05

**Authors:** Chunlin Ou, Zhenqiang Sun, Xiaoyun He, Xiaoling Li, Songqing Fan, Xiang Zheng, Qiu Peng, Guiyuan Li, Xiayu Li, Jian Ma

**Affiliations:** ^1^ Xiangya Hospital Department of Pathology Cancer Research Institute Central South University Changsha Hunan 410008 China; ^2^ Key Laboratory of Carcinogenesis and Cancer Invasion of Ministry of Education Central South University Changsha 410078 China; ^3^ NHC Key Laboratory of Carcinogenesis Central South University Changsha 410078 China; ^4^ Hunan Key Laboratory of Nonresolving Inflammation and Cancer Department of Gastroenterology The Third Xiangya Hospital Central South University Changsha 410013 China; ^5^ Department of Anorectal Surgery The First Affiliated Hospital of Zhengzhou University Zhengzhou Henan 450052 China; ^6^ Department of Pathology The Second Xiangya Hospital Central South University Changsha 410011 China

**Keywords:** colorectal cancer, FSCN1, Hippo pathway, LINC00152, YAP1

## Abstract

As a transcription coactivator, Yes‐associated protein 1 (YAP1)'s role in tumorigenesis is well established. However, the mechanism of YAP1‐regulating long noncoding RNAs (lncRNA) in tumors is still largely unknown. Here, a YAP1 target gene, long intergenic noncoding RNA 00152 (LINC00152), which is highly expressed in colorectal cancer (CRC), is identified. The oncogenic functions of LINC00152 in CRC are demonstrated by a panel of in vitro and in vivo experiments. Further studies reveal the potential downstream mechanisms of LINC00152, which can act as a competing endogenous RNA sponging with miR‐632 and miR‐185‐3p to regulate Fascin actin‐bundling protein 1 (FSCN1) expression and thus promote the malignant proliferation and metastasis in CRC cells. Targeting the YAP1/LINC00152/FSCN1 axis inhibits the progression of CRC. This finding provides a new regulatory model of the “YAP1‐lncRNA” in CRC, which gives rise to a new perspective, “YAP1/LINC00152/miR‐632‐miR‐185‐3p/FSCN1,” to explore the cancer‐promoting mechanism of YAP1 involved in CRC.

## Graphical abstract

Cancer‐associated long noncoding RNAs (lncRNAs) are emerging as critical regulators in tumorigenesis. Here, a Yes‐associated protein 1 (YAP1) target lncRNA LINC00152 is identified, and a critical role of YAP1/LINC00152/FSCN1 axis in the tumorigenesis of colorectal cancer (CRC) is revealed, which may provide a potential new target for CRC diagnosis and therapy.

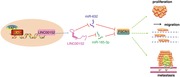

## 1 Introduction

With the quick development of high‐throughput RNA deep sequencing, more and more novel functional cancer‐associated long noncoding RNAs (lncRNAs) have been identified and validated,^1^, ^2^ including those in colorectal cancer (CRC). Well‐known CRC‐associated lncRNAs contain colorectal cancer‐associated lncRNA (CCAL),^3^ colon cancer‐associated transcript 1 (CCAT1) and colon cancer‐associated transcript 2 (CCAT2).^4^ Since lncRNAs regulate a subset of genes, and are transcriptionally regulated by a series of transcription factors, targeting these lncRNAs induces a much greater effect on cancer cells than targeting single gene.^5^ Therefore, exploring the upstream and downstream regulation mechanism of cancer‐associated lncRNAs has been gaining widespread attention.

As the main effector of the Hippo pathway, Yes‐associated protein 1 (YAP1) plays a key role in regulating multiple biological function, including cell–cell contact inhibition, proliferation, and differentiation.^6^, ^7^ As a transcription coactivator, YAP1 is abnormally expressed in various malignancies,^8^, ^9^ and modulates biological phenotypes of cancer cells via regulating a number of target genes, such as *CTGF*, *CYR61*, and *AREG*.^7^, ^8^ Recently, a novel regulatory model of YAP1 transcriptionally regulating the noncoding RNAs (ncRNAs) in CRC has attracted much attention, in which of these noncoding RNAs, including microRNAs (miR‐130a^10^ and miR‐29^11^), as well as lncRNAs (RMRP,^12^ BCAR4,^13^ MALAT1,^14^ and lncARSR^15^). However, the mechanism and function of YAP1 transcriptionally regulating lncRNAs in tumorigenesis still remains elusive.

We have recently shown that YAP1 is expressed in CRC at an enhanced level.^16^ Suppression of YAP1 in CRC cell line, HCT116, caused downregulation of 288 genes, and those genes are involved in malignant proliferation and epithelial‐mesenchymal transition (EMT), suggesting that YAP1 targeting genes play an important role in progression of CRC. However, that the differently expressed lncRNAs derive from those differently expressed genes is not explored in the previous study.^16^ Therefore, we studied the role of YAP1 targeting lncRNAs in the current study.

Here, we have characterized one of these YAP1‐targeting lncRNAs, LINC00152, which is expressed at high levels in human CRC tissues. Strikingly, suppression of LINC00152 caused downregulation of 159 genes, and as a result ceased the malignant proliferation, invasion and metastasis of CRC cells. LINC00152 binds several tumor suppressor microRNAs and inhibiting these microRNAs partially rescued LINC00152‐suppression cells from the inhibition of cell proliferation, invasion and metastasis. Consequently, establishing a new regulatory axis of the “YAP1‐LINC00152” could better to explore the cancer‐promoting mechanism of YAP1 in CRC.

## 2 Results

### 2.1 YAP1‐Associated LINC00152 Is Highly Expressed in Human CRC Tissues

**Figure 1:**
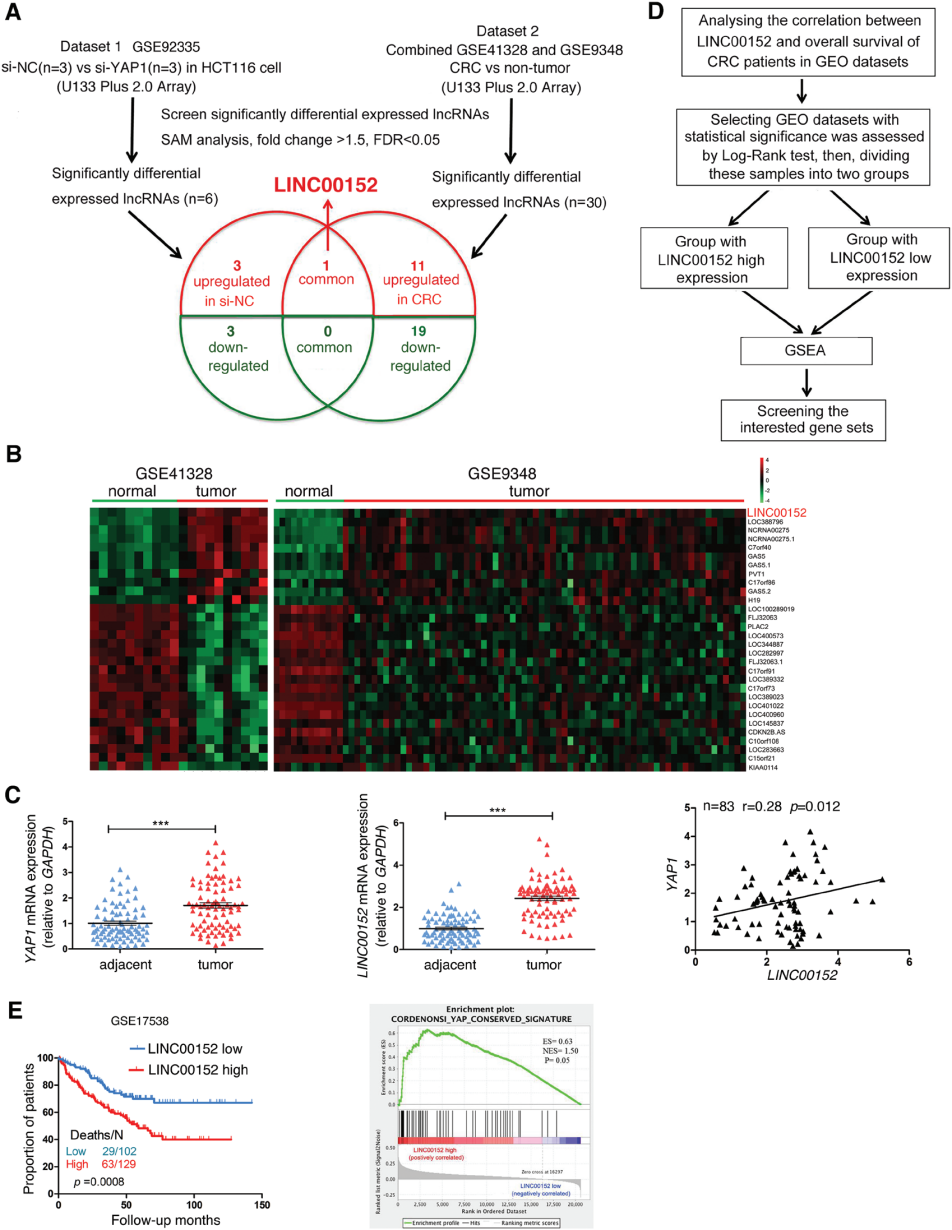
YAP1‐associated LINC00152 is highly expressed in human CRC tissues. A) Schematic overview of the workflow used to investigate the “YAP1‐lncRNAs” regulatory axis in CRC. We constructed a screening strategy via combination of two sets of gene expression profile date: one is to analyze the differentially expressed lncRNAs induced by si‐*YAP1* (#GSE92335, si‐*YAP1* vs si‐NC in colon cancer cells, to explore the downstream molecules of YAP1); the another is to analyze the differentially expressed lncRNAs between colorectal cancer tissues and normal colorectal tissues using two sets of CRC expression profiles data (#GSE41328 and GSE9348). B) Heatmap of 30 dysregulated lncRNAs mined from GSE41328 and GSE9348. C) Left and middle: The expression of *YAP1* and *LINC00152* were analyzed by RT‐qPCR in 83 pairs CRC samples and corresponding adjacent normal colorectal samples. Right: Correlation analysis of *YAP1* and *LINC00152* expression levels by Spearman's rank correlation coefficient. D) Schematic flowchart showing a strategy to analyze the gene set differences between *LINC00152* ^low^ and *LINC00152* ^high^ in CRC specimens from GEO database revealed by GSEA analysis. E) Left: Kaplan–Meier analysis shows overall survival (OS) curves of CRC patients with different expression of LINC00152, which statistical significance was assessed by log‐rank test (#GSE17538, the specimen was divided into two groups: group 1, *LINC00152* ^low^, *n* = 102; group 2, *LINC00152* ^high^, *n* = 129). Right: GSEA analysis showed the different gene set between *LINC00152* ^low^ and *LINC00152* ^high^. ES, enrichment score; NES, normalized enrichment score. ***p < 0.001.

To investigate the “YAP1‐lncRNAs” regulatory axis in CRC, we constructed a screening strategy (**Figure** **1**A) via combination of two sets of gene expression profile date: one is to analyze the differentially expressed lncRNAs induced by si‐*YAP1* in CRC cells (i.e., si‐*YAP1* vs si‐NC in colon cancer cells, to explore the downstream molecules of YAP1, see our previous study,^16^ #GSE92335) through significant analysis of microarray (SAM);^17^ the another is to analyze the differentially expressed lncRNAs between colorectal cancer biopsies and normal colorectal tissues using two sets of microarray data (#GSE41328 and GSE9348) (Figure 1B). LINC00152 was not only the most significantly decreased lncRNA after suppressing the *YAP1* expression in CRC cell line (Table S1, Supporting Information), but also the most significantly upregulated lncRNA in CRC datasets (Figure 1B; Tables S2 and S3, Supporting Information). Furthermore, *LINC00152* was also upregulated in CRC tissues in The Cancer Genome Atlas (TCGA) database and multiple gene expression omnibus (GEO) databases (Figure S1A,C, Supporting Information). Receiver operating characteristic (ROC) curve analysis was performed to evaluate the diagnostic values of LINC00152 for the TCGA CRC datasets, which was 0.91 with a 95% confidence interval of 0.86–0.96 (p < 0.001), as depicted in Figure S1A (right panel) in the Supporting Information.

We further validated *LINC00152* expression levels and the correlation between *YAP1* and *LINC00152* in another cohort of CRC samples using RT‐qPCR. YAP1 and LINC00152 were highly expressed in 83 cases CRC tissues compared with matched para‐tumor tissues, meanwhile, *LINC00152* expression was positively correlated with *YAP1* level (Figure 1C). Moreover, increased *LINC00152* expression in CRC tissues clearly correlated with a poor overall survival (OS) in CRC patients (Figure 1E, left panel). Gene set enrichment analysis (GSEA) revealed the “YAP conserved signature” gene sets is strongly enriched in *LINC00152* ^high^ CRC specimens compared with *LINC00152* ^low^ specimens (see the Experimental Section; Figure 1D,E, right panel). The expression of *LINC00152* was also positively correlated with *YAP1* and its target gene *CTGF* 18 in CRC samples (Figure S1B,D,E, Supporting Information). Collectively, these results demonstrated that LINC00152 is a downstream lncRNA of YAP1, and is highly expressed in human CRC tissues, predicting unfavorable prognosis.

### 2.2 YAP1 Transcriptional Regulates LIC00152 Expression in CRC Cells

**Figure 2:**
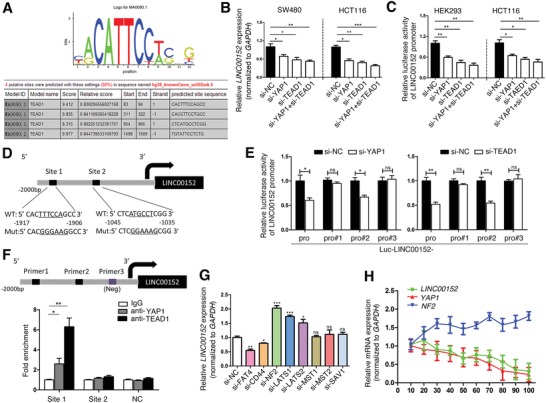
YAP1, as well other Hippo pathway molecules, transcriptional regulate LINC00152 expression. A) The TEAD1 binding motif in *LINC00152* predicted from JASPAR matrix models. B) RT‐qPCR analysis of *LINC00152* expression levels in CRC cells transfected with indicated siRNAs after 48 h. C) Cells were cotransfected with a luciferase reporter containing the *LINC00152* promoter (Luc‐LINC00152‐pro) and the indicated siRNAs for 36 h. D) A schematic diagram showing the human *LINC00152* upstream promoter region, including the predicted TEAD1‐binding regions (Site 1 and Site 2), as well as the wild‐type (WT) and two mutated (Mut) *LINC00152* promoter luciferase constructs. E) Promoter activities from HCT116 cells cotransfected with luciferase reporters of differently predicted binding‐site plasmids and treated with the indicated siRNAs, were measured by dual luciferase reporter assay. Luc‐ LINC00152‐pro luciferase reporter contains two WT binding sites; Luc‐LINC00152‐pro‐#1 contains the Site 1 Mut binding site and Site 2 WT binding site, Luc‐LINC00152‐pro‐#2 contains the Site 1 WT binding site and Site 2 Mut binding site, and Luc‐LINC00152‐pro‐#3 contains both the Site 1 and Site 2 Mut binding sites. F) Upper: Putative TEAD1‐binding sites on the promoter region of *LINC00152*, and design indicated primers. Lower: ChIP assays of the enrichment of YAP1 and TEAD1 on *LINC00152* promoter relative to control IgG in HCT116 cells. G) RT‐qPCR analysis of *LINC00152* expression levels in HCT116 cells transfected with indicated siRNA after 48 h. H) Dynamic change of expression levels of *NF2*, *YAP1*, and *LINC00152* under the influence of cell density change (from 10% to 100% confluence, x axis) were analyzed by RT‐qPCR. Data are shown as mean ± s.e.m. *p < 0.05, **p < 0.01, ***p < 0.001 compared with control.

To confirm the regulatory relationship between YAP1 and LINC00152 in CRC, we first demonstrated that *YAP1* overexpression or inhibition resulted in significant change of *LINC00152* expression levels (Figure S2, Supporting Information). Moreover, LINC00152 is required for YAP1‐induced cell proliferation and tumor growth of CRC (Figures S3A and S4, Supporting Information). We further explored the molecules mechanism by which YAP1 regulates LINC00152. As YAP1 cannot bind DNA directly and must interact with DNA‐binding transcription factors, hyperactivated YAP1 enters the nucleus to bind members of the TEA domain transcription factor (TEAD) family or other transcription factors to exert biological function. Therefore, we first used the bioinformatics analyses (JASPAR, ifti.org, and UCSC) and predicted that transcription factor TEAD1 has two binding sites for the *LINC00152* promoter, “CACTTTCCAGCC” (Site 1) and “CTCATGCCTCGG” (Site 2) (**Figure** **2**A,D). Meanwhile, correlation analysis indicated that *LINC00152* expression was positively related with *TEAD1* level (Figure S1F,G, Supporting Information). Suppression of *YAP1* or *TEAD1* inhibited *LINC00152* expression and its promoter activity (−2000 to +500 region of the *LINC00152* promoter) in CRC cells (Figure 2B,C; Figures S2 and S3B,C, Supporting Information). Furthermore, Figure 2E indicates suppression of *YAP1* or *TEAD1* reduced the luciferase activities of Luc‐152‐pro and Luc‐152‐pro‐#2, both containing the WT binding of Site 1 that was away from the start site of *LINC00152* transcription (Figure 2D). However, suppression of *YAP1* or *TEAD1* did not affect the activities of Luc‐152‐pro‐#1 and Luc‐152‐pro‐#3, both containing the mutation binding of Site 1 (see Figure 2D,E legend for detail). This suggests that TEAD1 can regulate the expression of *LINC00152* by interacting with the binding Site 1 in the promoter region of *LINC00152*. chromatin immunoprecipitation (ChIP)‐qPCR further vilified that TEAD1 and YAP1 was found to binding to the Site 1 promoter sequence of *LINC00152* (Figure 2F). These findings revealed that YAP1 can transcriptionally regulate the expression of *LINC00152* with the help from TEAD1.

As YAP1 is the main effector of the Hippo pathway, we investigated whether the upstream molecules (e.g., CD44, FAT4, and NF2) and core kinase (e.g., LATS1/2, MST1/2, and SAV1) of the Hippo pathway also modulate the expression of *LINC00152*. We found that tumor‐suppressor NF2 negatively regulate the *LINC00152* expression and promoter activity (Figure 2G; Figure S5A–C, Supporting Information). Interestingly, NF2 is a sensor in the cell–cell contact inhibition of the Hippo signaling.^19^, ^20^ We thus investigated whether cell density change could modulate *LINC00152* expression in the previous conducted model of different culture cell confluence (i.e., from 10% to 100%).^16^ Figure 2H demonstrates that the expression of *NF2* was gradually increased along with cancer cell density arising, while *LINC00152* and *YAP1* expression was decreased when cell density was arising. We next asked whether the cancer cell density‐YAP1‐LINC00152 axis is dependent on NF2 expression. Figure S5D (Supporting Information) shows that siRNA‐*YAP1*, as well as *NF2* overexpression, can reduce the scale of *LINC00152* response to cell density change. The nuclear expression of *LINC00152* in high cell density was lower than that in low cell density, whereas the cytoplasm expression of *LINC00152* had the opposite tendency (Figure S5E, Supporting Information), which may imply the degradation of LINC00152 mainly happens in the cytoplasm. These findings suggested that LINC00152 is a cell density‐sensitive lncRNA; YAP1, as well other Hippo pathway molecules, transcriptional regulates LINC00152 expression in CRC cells.

### 2.3 LINC00152 is an Oncogenic lncRNA in CRC Cells

**Figure 3:**
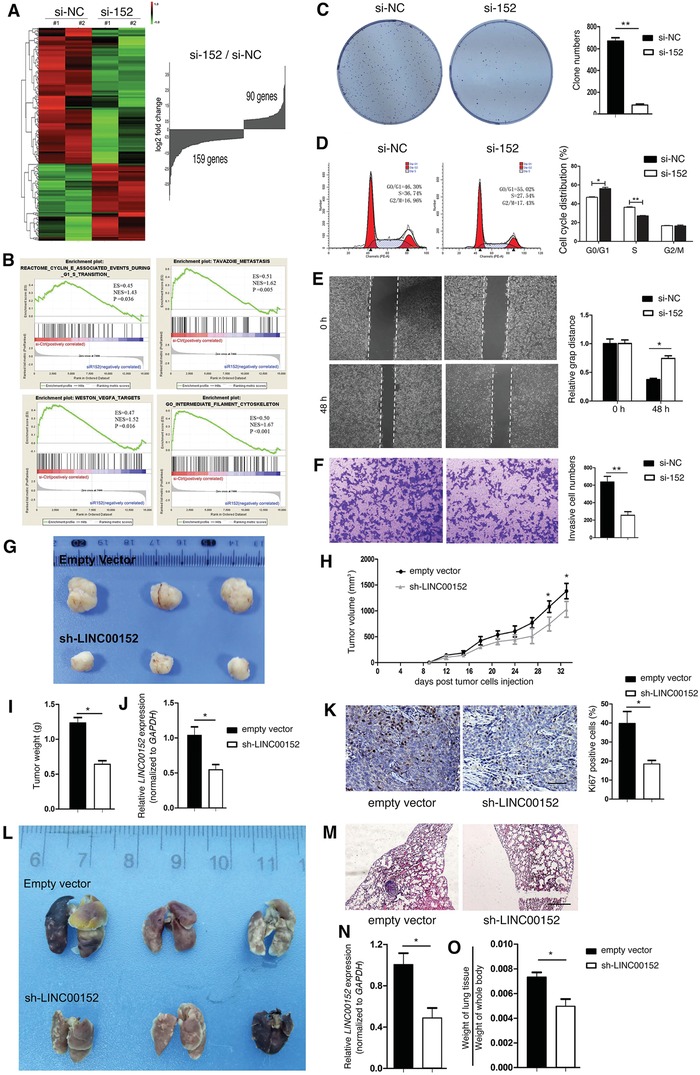
*LINC00152* is an oncogenic lncRNA in CRC cells. A) Left: Heat map showing the differential expressed genes induced by *LINC00152* siRNA in HCT116 cells. Red and Green indicate high and low mRNA expression levels, respectively. Right: Compared to control group (si‐NC), si‐*LINC00152* (si‐152) upregulated 90 genes, whereas downregulated 159 genes expression levels (fold change > 1.5). B) Correlation analysis between *LINC00152* and cancer‐associated gene sets, as demonstrated by GSEA (analyzing si‐NC vs si‐152 HCT116 cells). ES, enrichment score; NES, normalized enrichment score. C–F) HCT116 cells were transfected with indicated si‐NC or si‐152 for 24 or 48 h. The cell proliferative ability was determined by cell clone‐formation assay (C), and cell cycle analysis (D); the cell migration and invasion abilities were determined by wound‐healing assay (E), and transwell matrigel assay (F). G–K) Five‐week‐old male nude mice were randomly divided into two groups: empty vector group and sh‐*LINC00152* group. Tumors were formed after HCT116 cells (stably transfected with the empty vector or sh‐LINC00152 vector) were subcutaneously injected into the flanks of nude mice. Excised xenograft tumors were measured 27 days after HCT116 cells injection for tumor size (G,H) and weight (I). J) RT‐qPCR results show *LINC00152* mRNA levels in tumor tissue specimens from engrafted nude mice. K) Immunohistochemical staining show Ki‐67 expression patterns in excised tumors. L–O) HCT116 cells (stably transfected with the empty vector or sh‐LINC00152 vector) were injected into the nude mice through tail vein of nude mice. 60 days later, the mice were euthanized, and metastatic lung nodules were detected macroscopically (L). M) Representative microscopic images of pulmonary metastatic lesions in two group nude mice. Original magnification, 40×. Scale bar: 200 µm. N) Expression levels of *LINC00152* in metastatic lung nodules of nude mice were determined by RT‐qPCR. O) The ratio of weight of lung tissue to weight of whole body reflected the metastasis ability of HCT116 cells in vivo. Data are shown as mean ± s.e.m. *p < 0.05, **p < 0.01 compared with control. Each group has eight mice.

To investigate the molecular mechanism by which *LINC00152* is associated with CRC progression, we explored the gene expression profiles change of CRC cells upon *LINC00152* suppression. The HCT116 cells were treated with specific siRNAs targeting *LINC00152*, and the globe mRNA profiles were measured by RNA sequencing (**Figure** **3**A; Table S4, Supporting Information). The top 20 changed pathways predicted by the differently expressed genes were listed in Table S5 (Supporting Information), which suggested there are a panel of key pathways that are affected by *LINC00152* suppression, including colorectal cancer signaling pathway, adherent junction, and apoptosis, etc. GSEA on the mRNA profiles change revealed positive associations between LINC00152 and multiple gene sets, including gene sets involved in cell cycle, metastasis, cytoskeleton, and vascular endothelial growth factor A signaling (Figure 3B). Likewise, the differently gene sets between *LINC00152* ^high^ and *LINC00152* ^low^ CRC specimens, are also involved in EMT, extracellular matrix pathways, etc. (Figure S6, Supporting Information). These findings suggested that LINC00152 may play an important role in CRC tumorigenesis and metastasis.

We asked whether LINC00152 is an oncogenic lncRNA in CRC cells. siRNA mediated *LINC00152* inhibition in HCT116 cells markedly decreased cell proliferative and invasive abilities (Figure 3C–F; Figure S3A, Supporting Information); and inhibited the expression of mesenchymal markers (Vimentin and β‐catenin) and G1‐S transition‐promoting markers (Cyclin D1 and CDK4), whereas increased the expression of epithelial marker (E‐cadherin) and cell cycle G1‐S transformation‐inhibiting marker (p27) (Figure S7, Supporting Information). We constructed two stable cell lines using shRNA vector to mediate *LINC00152* suppression in HCT116 cells, designated as empty vector and sh‐*LINC00152*. In the xenograft nude mouse model, sh‐LINC00152 significantly reduced the growth of xenograft tumors (Figure 3G–K); and in the “tail vein–lung metastasis” nude mouse model, sh‐*LINC00152* significantly reduced the formation of pulmonary metastatic nodules (Figure 3L–O). Collectively, these results demonstrated that LINC00152 is an oncogenic lncRNA, and promoted the tumorigenesis and metastasis of CRC cells both in vitro and in vivo.

### 2.4 LINC00152 Targets FSCN1 by Sponging with miR‐185‐3p and miR‐632

**Figure 4:**
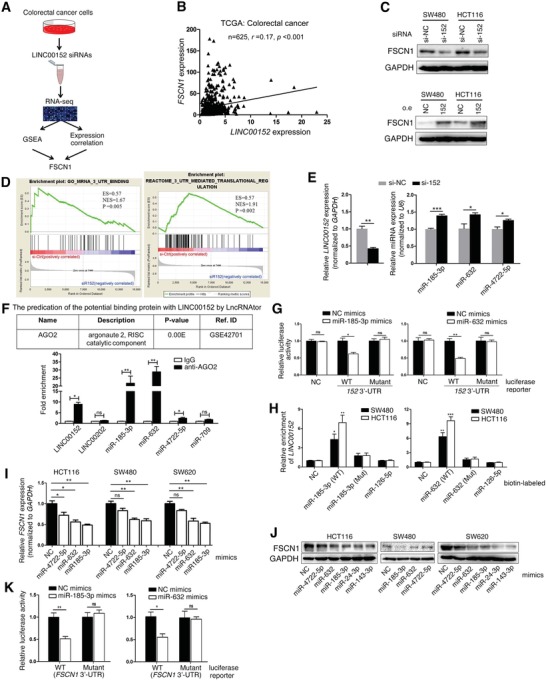
LINC00152 targets FSCN1 by sponging with miR‐185‐3p and miR‐632. A) Schematic flowchart showing the results of a transcriptome sequencing study on LINC00152‐associated pathways. CRC cells were treated with siRNAs for *LINC00152* or control siRNAs, and the mRNA expression profiles were determined. The combination of GSEA and gene expression correlation analysis identified FSCN1 in the cell metastasis and proliferation as a potential regulatory target of LINC00152. B) Correlation analysis between the mRNA expression levels of *LINC00152* and *FSCN1* in CRC tissues from TCGA data. C) Western blotting to measure FSCN1 protein levels in SW480 and HCT116 cells transfected with indicated siRNAs for 48 h. D) Correlation analysis between *LINC00152* and mRNA 3′‐UTR‐associated gene sets, as demonstrated by GSEA. E) RT‐qPCR to measure indicated RNAs levels in HCT116 cells transfected with si‐NC or si‐152 for 48 h. F) Upper: The predication of the potential binding protein with LINC00152 by LncRNAtor; lower: AGO2‐RIP assay was performed in HCT116 lysates, followed by RT‐qPCR to detect LINC00152 and indicated microRNAs associated with AGO2. G) Luciferase activity in HCT116 cells cotransfected with indicated mimics and luciferase reporters containing WT or mutant LINC00152 3′‐UTR. H) An RNA pull‐down assay followed by biotin‐labeled miR‐185‐3p and miR‐632 (WT or mutant) to detect whether LINC00152 endogenously associates with miR‐185‐3p and miR‐632. I–J) RT‐qPCR and Western blotting to measure FSCN1 mRNA and protein levels in CRC cells transfected with indicated mimics. K) Luciferase reporter constructs containing WT or mutated *FSCN1* 3ʹ‐UTRs were cotransfected with indicated mimics into HCT116 cells for 36 h. Relative firefly luciferase expression was normalized to Renilla luciferase. Data are shown as mean ± s.e.m. *p < 0.05, **p < 0.01, and ***p < 0.001 compared with control.

By analysis of the correlation between LINC00152 and mRNAs from our RNA sequencing dataset (Figure 3A), we found that Fascin actin‐bundling protein 1 (FSCN1) was assigned the obviously correlation coefficient *LINC00152*‐associated pathways (schematic shown in **Figure** **4**A). Fascin organizes F‐actin into parallel bundles and is required for the formation of actin‐based cellular protrusions, playing an important role in cell migration and motility.^21^, ^22^ FSCN1 was highly expressed in CRC cells compared with that in normal colon mucosal cells (Figure S8, Supporting Information) and promoted the malignant proliferation, migration, and invasion of CRC cells (Figures S3D and S9, Supporting Information). Based on these results, we proposed that a correlation might exist between LINC00152 and FSCN1. TCGA CRC database revealed that *LINC00152* expression positively correlated with *FSCN1* levels in the CRC tissues (Figure 4B). Suppression or overexpression of *LINC00152* significantly reduced or increased the level of FSCN1 (Figure 4C; Figure S10, Supporting Information). These results indicated FSCN1 maybe a target of LINC00152.

We next explore the mechanism for LINC00152 regulating the expression of *FSCN1*. LncRNAs can function as microRNA sponges in the cytoplasm or regulate gene expression in the nucleus as nuclear transcriptional regulators.^23^, ^24^ As shown in Figure S11 (Supporting Information), LINC00152 was mainly localized in the cytoplasm of CRC cells. Meanwhile, GSEA demonstrated positive associations between LINC00152 and the gene sets “mRNA 3ʹ‐untranslated regions (UTR) binding” and “3ʹ‐UTR mediated translational regulation” (Figure 4D). Therefore, we searched for microRNA‐binding sites both in the *LINC00152* sequence and 3ʹ‐UTR of *FSCN1* via bioinformatics analysis, and it showed that both *LINC00152* and 3′‐UTR of *FSCN1* contains sequences complementary to miR‐185‐3p, miR‐632, miR‐4722‐5p (Figure S12, Supporting Information), and these molecules expression were regulated by si‐*LINC00152* (Figure 4E) or si‐*Dicer1* (Figure S13, Supporting Information). To determine whether LINC00152 and miR‐185‐3p, miR‐632, miR‐4722‐5p are in the same RNA‐induced silencing complex (RISC), we performed an RIP assay. The level of LINC00152 and miR‐185‐3p, miR‐632, and miR‐4722‐5p were higher in the anti‐Ago2 group than that in the antinormal IgG group (Figure 4F). Among the three candidate miRNAs, miR‐185‐3p and miR‐632 showed more significant regulatory effect to FSCN1. Meanwhile, miR‐185‐3p and miR‐632 expression levels were lower in CRC tissues compared with that in the matched para‐tumor tissues, and negatively correlated with *LINC00152* and *FSCN1* levels (Figure S14, Supporting Information). Since miR‐4722‐5p expression levels showed no difference between CRC and matched para‐tumor tissue (Figure S14A, right, Supporting Information), we had not studied it further. On the one hand, both miR‐185‐3p and miR‐632 decreased the luciferase activity of the wild‐type *LINC00152* 3′‐UTR reporter vector, but not the mutant reporter vector (Figure 4G); additionally, biotin‐labeled miR‐185‐3p or miR‐632 (containing the wild‐type or site‐specific mutant) were used to pull‐down LINC00152, and *LINC00152* levels were significantly elevated in the complexes sedimented by the wild‐type miR‐185‐3p or miR‐632, but not by the mutant miR‐185‐3p or miR‐632 (Figure 4H). On the other hand, overexpression of miR‐185‐3p or miR‐632 reduced both mRNA and protein expression levels of FSCN1 in CRC cells (Figure 4I,J); additionally, miR‐185‐3p or miR‐632 significantly reduced the luciferase activities of wild‐type 3′‐UTR of *FSCN1*, but not the mutant 3′‐UTR of *FSCN1* (Figure 4K). These results indicated that LINC00152 targets FSCN1 by sponging with miR‐185‐3p and miR‐632.

### 2.5 LINC00152‐miR‐185‐3p/632‐FSCN1 Axis Promotes the Tumorigenesis of CRC

**Figure 5:**
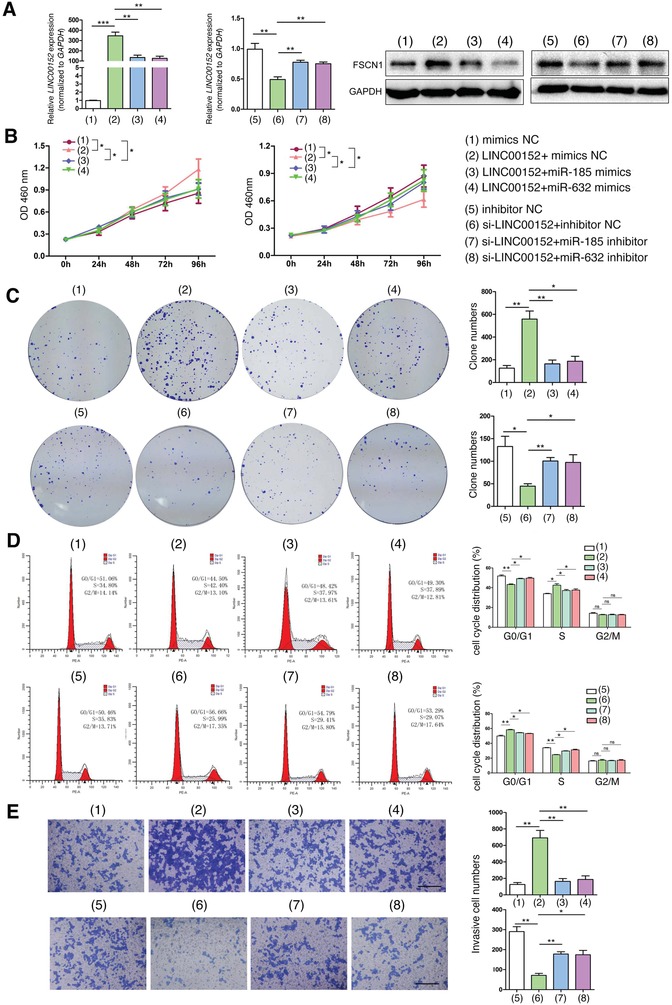
LINC00152‐miR‐185‐3p/632‐FSCN1 axis promotes the tumorigenesis of CRC in vitro. HCT116 cells were transfected with indicated mimics/inhibitors, siRNAs or plasmids for 24 or 48 h. A) The *LINC00152* mRNA and FSCN1 protein levels were determined by RT‐qPCR and Western blotting, respectively. The cell proliferative ability was determined by B) CCK8 assay, C) cell clone‐formation assay, and D) cell‐cycle analysis. E) The cell invasive ability was determined by transwell matrigel assay. Data are shown as mean ± s.e.m. *p < 0.05, **p < 0.01, and ***p < 0.001 compared with control.

In light of the above findings, we hypothesized that LINC00152/miR‐632‐miR‐185‐3p/FSCN1 axis might play a role in the tumorigenesis of CRC. HCT116 cells were transfected with different vectors/mimics/siRNAs as indicated in **Figure** **5**A,B. We validated different cell groups for in vitro experiment as indicated in Figure 5. We found that *LINC00152* overexpression increased cell proliferative and invasive abilities, whereas miR‐185‐3p and miR‐632 mimics markedly rescued theses phenotypes. In contrast, *LINC00152* suppression decreased cell proliferative and invasive abilities, whereas miR‐185‐3p and miR‐632 inhibitors markedly rescued theses phenotypes (Figure 5B–E).

**Figure 6:**
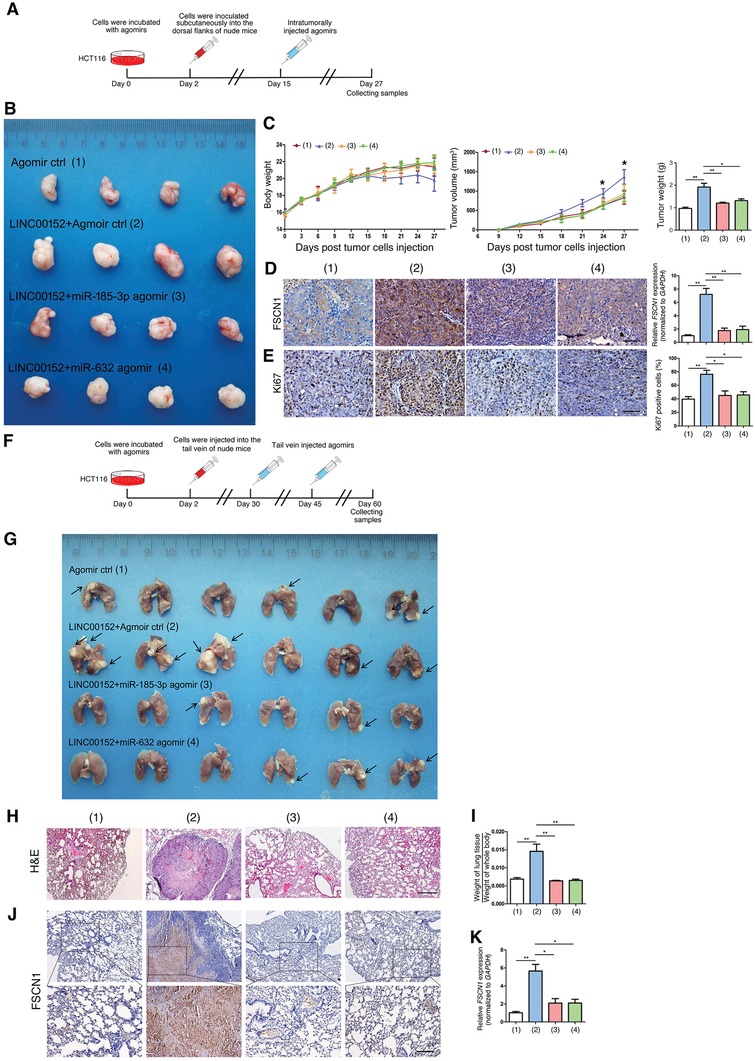
LINC00152‐miR‐185‐3p/632‐FSCN1 axis promotes the tumorigenesis of CRC in vivo. Five‐week‐old male nude mice were randomly divided into four groups: 1) agomir negative control, 2) LINC00152 overexpression + agomoir negative control, 3) LINC00152 overexpression + miR‐185‐3p agomir, 4) LINC00152 overexpression + miR‐632 agomir. A) Schematic illustration of agomirs injection for the subcutaneous xenograft nude mice model. B) Comparison of tumor engraftment size and weight in nude mice subcutaneously injected into the flanks with HCT116 cells transfected with indicated agomirs or expression plasmids. C) Left: The body weight of the four groups of nude mice. Middle: The mice xenograft tumor growth curves of the four groups. Right: The tumor weight of the four groups. D) Left: FSCN1 protein expression patterns within the tumor tissues from the nude mice were assayed by immunohistochemical staining. Right: *FSCN1* mRNA levels within the tumor tissues from the nude mice were assayed by RT‐qPCR. E) Immunohistochemical staining for quantification of the proliferation marker Ki67 in the tumor tissues. F) Schematic illustration of agomirs injection for the “tail vein–lung metastasis” nude mice model. G) After the injection of HCT116 cells transfected with indicated agomirs or expression plasmids into the tail vein of nude mice at 60 days, the mice were euthanized, and metastatic lung nodules were detected macroscopically. H) Representative microscopic images of pulmonary metastatic lesions in four group nude mice. I) The ratio of weight of lung tissue to weight of whole body reflected the metastasis ability of HCT116 cells in vivo. J) Immunohistochemistry detecting the staining of FSCN1 proteins in metastatic lung nodules of nude mice. Upper: Original magnification, 40×. Scale bar: 200 µm; lower: original magnification, 200×. Scale bar: 50 µm. K) mRNA expression of *FSCN1* in metastatic lung nodules of nude mice were determined by RT‐qPCR. Data are shown as mean ± s.e.m. *p < 0.05 and **p < 0.01 compared with control.

We next investigated the role of the axis in CRC development by means of xenograft nude mouse model (**Figure** **6**A–E) and “tail vein–lung metastasis” nude mouse model (Figure 6F–K). The nude mice were randomly divided into four groups as indicated in Figure 6B, G. *LINC00152* overexpression vector significantly increased the growth of xenograft tumors and lung tumorigenicity of metastatic tumors in nude mice, whereas miR‐185‐3p and miR‐632 agomirs treatment can largely reverse these phenotypes (Figure 6). The tumor tissues showed that FSCN1 protein expression levels were increased upon *LINC00152* overexpression, whereas miR‐185‐3p and miR‐632 agomirs treatment can reverse this effect (Figure 6D,J,K). Collectively, these results demonstrated that LINC00152/miR‐632‐miR‐185‐3p/FSCN1 axis promotes the proliferation, invasion, and metastasis of CRC cells both in vitro and in vivo.

### 2.6 LINC00152 and FSCN1 Are Associated with CRC Clinicopathologic Factors

**Figure 7:**
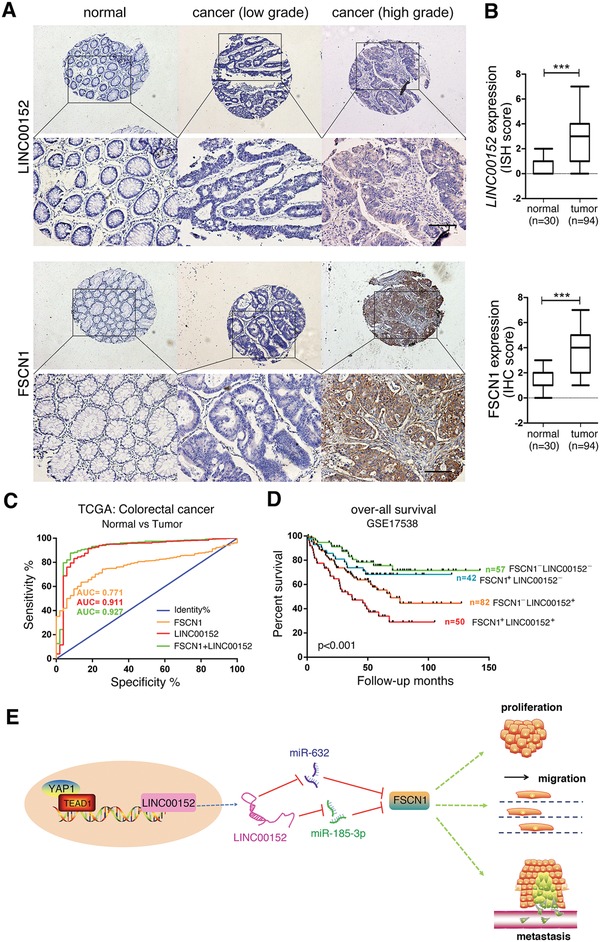
LINC00152 and FSCN1 expression levels are associated with clinicopathological parameters of CRC patients. A) In situ hybridization and immunohistochemistry staining detection of *LINC00152* RNAs and FSCN1 proteins in CRC tissue microarray, respectively. Upper: Original magnification, 40×. Scale bar: 200 µm; Lower: original magnification, 200×. Scale bar: 50 µm. B) Expression scores of *LINC00152* RNAs and FSCN1 proteins in CRC tissue microarray that was containing 30 cases normal mucosa tissues and 94 CRC tumor tissues. C) Receiver‐operating characteristic (ROC) curves displaying the sensitivity and specificity of *LINC00152* or FSCN1 combined with *LINC00152* expression for the diagnosis of CRC patients from TCGA data. Insets indicate AUC values, 95% confidence intervals, and statistics. D) Overall survival (OS) according to Kaplan–Meier analysis shows a difference in the survival between CRC patients with overexpression of both LINC00152 and FSCN1 as compared with the lower expression of both transcripts in the GSE17538 dataset. E) Schematic representation of a model for the major molecular mechanisms of “YAP1/LINC00152/miR‐632‐miR‐185‐3p/FSCN1” axis in CRC. ***p < 0.001.

To investigate the clinical significance of *LINC00152* and FSCN1 in CRC specimens, we examined *LINC00152* and FSCN1 expression levels by analyzing tissue microarray containing 30 cases normal colorectal mucosa tissues and 94 CRC tumor tissues. Strong positive expression of *LINC00152* and FSCN1 proteins were identified in the cytoplasm of cancer tissues, meanwhile, weak staining was observed in normal tissues (**Figure** **7**A,B). Statistical analysis revealed that *LINC00152* expression levels positively correlated with FSCN1 levels in the CRC tissues (**Tables** **1** and **2**), which were consistent with date from TCGA database (Figure 4B; Figure S15A, left, Supporting Information).

**Table 1:** Correlation between LINC00152 or FSCN1 expression and clinicopathological parameters in 94 cases of CRC cancer tissues (χ^2^ test)

	*n*	LINC00152				FSCN1			
		Low (36)	High (58)	χ^2^	*p*	Low (28)	High (66)	χ^2^	*p*
Age				0.471	0.492			0.590	0.443
<60	46	16	30			12	34		
≧60	48	20	28			16	32		
Gender				0.085	0.770			0.763	0.382
Male	54	20	34			18	36		
Female	40	16	24			10	30		
Tumor location				2.495	0.114			3.472	0.062
Colon	40	19	21			16	24		
Rectal	54	17	37			12	42		
TNM stage				8.223	0.004^a)^			7.983	0.005^a)^
I/II	41	9	32			6	35		
III/IV	53	27	26			22	31		
Lymph node metastasis				4.098	0.043^a)^			4.305	0.038^a)^
Yes	49	14	35			10	39		
No	45	22	23			18	27		

Note: Differences among variable were assessed by the χ^2^ test.

^a)^ The value had statistical significant differences.

Abbreviation: TNM, tumor node metastasis.

**Table 2:** Correlation of LINC00152 and FSCN1 expression in CRC cancer tissues (*n* = 94)

	LINC00152 expression (ISH)			
	Low (36)	High (58)	χ^2^	*p*
FSCN1 expression (IHC)			5.993	0.014
Low (28)	16	12		
High (66)	20	46		

Note: Differences among variable were assessed by the χ^2^ test.

Abbreviation: IHC, immunohistochemistry; ISH, in situ hybridization.

Univariate analysis showed that *LINC00152* expression was significantly associated with tumor size, tumor grade, tumor node metastasis (TNM) stage and distant metastasis (*p* < 0.05; Table 1). Meanwhile, FSCN1 expression levels were significantly associated with tumor size, TNM stage and distant metastasis (*p* < 0.05; Table 1). ROC curve analysis indicated that *LINC00152* combined with FSCN1 for the diagnosis was prior than the single indicator (Figure 7C; Figure S15B, Supporting Information). We also calculated OS for CRC patients by means of the combined index of *LINC00152* and FSCN1 expression (Figure 7D; Figure S15C,D, Supporting Information). CRC patients tended to have the worst prognosis if the expression of both *LINC00152* and FSCN1 was high, whereas this prognosis was intermediate if only one of the expression variants was upregulated and the other was downregulated. Optimal outcomes were observed if the expression of both variants was low. The human CRC clinical data supported the concept that LINC00152 and FSCN1 both has oncogenic function.

## 3 Discussion

YAP1 acts as a transcriptional coactivator interacting with the corresponding transcription factors to activate downstream mRNA and lncRNAs expression. For example, YAP1 interacts with TEAD1 bound to the lncRNA BCAR4 promoter, forming the YAP1‐BCAR4 axis which plays an oncogenic role in breast cancer development.^13^ Our previous study demonstrated the potential function and mechanism of YAP1 in tumorigenesis of CRC.^16^ In this study, we further explored the underlying downstream mechanism of YAP1 in CRC by constructing and screening the “YAP1‐lncRNA” network and identify a novel YAP1 target lncRNA, LINC00152. To the best of our knowledge, this is the first study to systematically evaluate the role of “YAP1‐lncRNA” network in CRC. We next used the luciferase and ChIP experiments to explore a direct binding between the promoter of *LINC00152* and YAP1 interacting with TEAD1, which provides evidence that LINC00152 is transcriptionally regulated by YAP1/TEAD1.

As the main effector of Hippo pathway, YAP1 responds to a wide range of extracellular and intracellular signals, including cell–cell contact,^20^ cell polarity,^25^ mechanical cues,^26^ ligands of G‐protein‐coupled receptors^27^ and cellular energy status.^28^ Regulation of YAP1 by cell density suggests a critical role for the Hippo pathway in contact inhibition, tissue growth, and tumorigenesis.^7^ Meanwhile, many established cancer cell lines exhibit growth in vitro that is impervious to contact inhibition, implying dysfunction of Hippo/YAP signaling. However, few studies report cell density could regulate the expression of lncRNAs. Therefore, we asked whether cell density could regulate the “YAP1‐LINC00152” axis via inducing upstream molecules of Hippo/YAP signaling dysfunction in CRC. Interestingly, we found the “YAP1‐LINC00152” axis was regulated by “cell density‐NF2” signaling cascade in CRC cells, forming a “cell density‐NF2‐YAP1‐LINC00152” axis.

LINC00152 is a large intergenic noncoding RNA (LincRNA) with a length of 852 bp, located at the location of chromosome 11.2 of genome 2 (87 455 455 to 87 521 518).^29^ Recent studies have shown that LINC00152 is highly expressed in a variety of tumors, such as, glioma,^30^ nonsmall cell lung cancer,^31^ gastric cancer,^32^ etc. Upregulated *LINC00152* could interact with many signaling pathways, thereby promote tumor cell proliferation, inflammatory response, invasion and metastasis.^33^, ^34^, ^35^, ^36^ In this study, we further investigated the potential function and mechanism of LINC00152 in tumorigenesis of CRC by RNA‐seq analysis, which revealed that LINC00152 might regulate TNF signaling pathway, B cell receptor signaling pathway, colorectal cancer, tight junction, and apoptosis, etc. (Table S5, Supporting Information), confirming LINC00152's importance in CRC tumorigenesis. Moreover, our study identified a novel LINC00152 target gene, FSCN1, which was also known as Fascin1 protein. Similar to the roles of LINC00152 in tumors, FSCN1 is not only highly expressed in a variety of tumors, including CRC,^37^ but also promotes the proliferation, invasion and metastasis of tumors.^38^, ^39^

LncRNAs display enormous variations in expression levels and show diversity in subcellular localizations.^21^ Increasing evidence showed that a novel regulatory mechanism existed between lncRNAs and microRNAs. lncRNAs can act as endogenous molecular sponges to compete for microRNAs, thereby negatively regulating microRNA expression.^40^ For example, in CRC, Chen et al.^41^ reported that the lncRNA UICLM can function as a competing endogenous RNA (ceRNA) for miR‐215 to regulate the expression of ZEB1; Li et al.^42^ demonstrated lncRNA ZFAS1 functions as an oncogene in hepatocellular carcinoma progression by binding miR‐150 and abrogating its tumor‐suppressive function. Since LINC00152 mainly localized in the cytoplasm of CRC cells (Figure S11, Supporting Information), we considered the mechanism that LINC00152 regulates FSCN1 may be as a ceRNA sponging with microRNAs. We identified and verified miR‐185‐3p and miR‐632 could bind to both LINC00152 and FSCN1. Previously, miR‐185‐3p and miR‐632 had been identified as tumor suppressors in many tumor types.^43^, ^44^ These data strongly suggested that LINC00152 acts as a ceRNA for miR‐185‐3p and miR‐632 to regulate FSCN1 expression in CRC cells.

Since regulatory RNAs such as lncRNA or microRNA regulate a subset of genes, targeting these noncoding RNAs induces a much greater effect on cancer cells than targeting single gene.^5^ In recent years, noncoding RNA was considered as a kind of therapeutic targets for tumors. Many targeting microRNAs' tumor drugs have entered the stage of clinical trials, for instance, MRX34 (NCT01829971, targeting miR‐34),^45^ Miravirsen (SPC3649, targeting miR‐122).^46^, ^47^ However, the research on targeting lncRNAs' tumor drugs is still in the stage of animal models, for example, by means of MMTV‐PyMT‐induced breast cancer mouse model, Arun et al.^48^ showed MALAT1 antisense oligonucleotides can slow down tumor growth and metastasis and inhibit breast cancer progression in vivo. In our study, we demonstrated that miR‐185‐3p and miR‐632 agomirs treatment could inhibit the tumorigenesis of CRC in xenograft nude mouse model and “tail vein–lung metastasis” model (Figure 6). However, we had not dissected the immune mechanism behind the “YAP1‐LINC00152‐FSCN1” axis's oncogenic phenotypes since nude mice were used in the in vivo study.

In conclusion, we identified a new YAP1 target lncRNA, LINC00152, which promoted the biological characteristics of CRC cells by sponging miR‐185‐3p and miR‐632 for upregulating its target FSCN1, as an “YAP1/LINC00152/FSCN1” axis to promote the malignant proliferation, migration and metastasis in CRC (Figure 7E). Through exploring the upstream and downstream regulatory mechanism of LINC00152, this study provided new insight into the potential use of YAP1/LINC00152/FSCN1 for the development of new treatment strategies for CRC.

## 4 Experimental Section

*Human Tissue Samples*: Two sets of human CRC samples were collected for this study: Set 1 contained 83 pairs of CRC tissues and corresponding adjacent normal mucosa tissues to verify *YAP1* and *LINC00152* expression with RT‐qPCR; and Set 2 included 94 paraffin‐embedded CRC and 30 normal colorectal mucosa tissue samples to detect the expression of *FSCN1* and *LINC00152* (Tables 1 and 2). The samples were compiled into a tissue microarray as previously described.^49^ All the human tissues were collected from the Second Xiangya Hospital of Central South University (Changsha, China). All specimens were confirmed by histopathological examination. Written informed consent was obtained from all study participants. Collections and use of tissue samples were approved by the ethical review committees of the Second Xiangya Hospital of Central South University and were in accordance with the Declaration of Helsinki.

*Bioinformatics Analysis*: Two independent cohorts of primary CRC data and their correlated clinic data, GSE9348^50^ and GSE41328,^51^ were downloaded from the Gene Expression Omnibus (GEO) database. GSE9348 has 70 primary colorectal cancer samples and 12 normal colorectal samples; GSE41328 contains ten pairs of colorectal cancer and adjacent nontumor tissues, respectively. GSE9348 and GSE41328 gene expression profiles date was analyzed by means of Significant Analysis of Microarray (SAM) software. The cutoff fold change for differentially expressed lncRNAs was set at ≥1.5, and the false discovery ratio was <5%. The significant differently expressed lncRNAs between CRC and nontumor tissues were shown by a heat map (Figure 1B) generated using Genesis software.

GSE17538^52^ contains the clinical follow‐up data of 231 CRC patients. Based on the results of the log‐rank test, the GSE17538 was divided into the low LINC00152 expression group (LINC00152^low^, *n* = 102) and high LINC00152 expression group (LINC00152^high,^ *n* = 129), then the gene set enrichment analysis (GSEA)^53^ was used to identify gene set differences between the two groups (LINC00152^low^ vs LINC00152^high^; Figure 1D).

*RNA Sequencing Analysis*: HCT116 cells were transfected with a scramble siRNA (si‐NC) or siRNA targeting *LINC00152* (si‐152) for 36 h. Total RNAs were extracted and detected by the Solexa high‐throughput sequencing service (Oebiotech, Shanghai, China) as described.^54^ Two independent biological replicates were plated. The RNA‐seq raw expression files and details have been deposited in NCBI GEO under accession no. GSE132519. Differentially expressed genes were identified using the DESeq (2012) functions estimateSizeFactors and nbinomTest.^55^ Log‐fold changes of up‐ or downregulated mRNAs between the two groups were selected with a significance threshold of *p* < 0.05. Only genes with greater than twofold change and *p*‐values of less than 0.05 were selected for pathway analysis by the Ingenuity Pathway Analysis system (https://www.ingenuity.com). Gene ontology (GO) enrichment and kyoto encyclopedia of genes and genomes pathway enrichment analysis of differently expressed genes were, respectively, performed using R based on the hypergeometric distribution. Hierarchical cluster analysis of differently expressed genes was performed to explore genes expression pattern. GSEA was used to compare the gene sets difference between the two groups (si‐NC vs si‐152).

*Cell Cultures, Plasmids, and Transfection*: HEK293 and HCT116 cells were maintained in DMEM (Dulbecco's modified Eagle's medium) with 10% fetal bovine serum (FBS, Invitrogen, Carlsbad, CA, USA), and other lines (SW480, HT29, NCM460, SW620, and CaCO_2_) were cultured in RPMI‐1640 media supplemented with 10% FBS. Transfection was conducted with Lipofectamine 3000 (Invitrogen) according to the manufacturer's instructions. The psiCHECK2‐LINC00152 promoter reporter (−2000 to +500 region of the LINC00152 promoter) plasmid and the corresponding mutant (psiCHECK2‐LINC00152‐pro‐#1, psiCHECK2‐LINC00152‐pro‐#2, psiCHECK2‐LINC00152‐pro‐#3) were purchased from Vigene (Rockville, NZ, USA). The psiCHECK2‐LINC00152‐Luc wild‐type (WT), psiCHECK2–LINC00152‐Luc MT (miR‐185‐3p), psiCHECK2–LINC00152‐Luc MT (miR‐632), psiCHECK2‐FSCN1‐3′‐UTR WT plasmids and the corresponding mutants were also purchased from Vigene. The pEGEP‐C1‐LINC00152 and the parental pEGEP‐C1 vector plasmids were obtained from GenePharma (Shanghai, China). microRNA mimics, inhibitors, and siRNAs were purchased from Ribobio (Guangzhou, China), and the sequences of siRNAs are shown in Table S6 (Supporting Information).

*Quantitative Real‐Time PCR*: Total RNA was isolated with Trizol (Life Technologies, Gaithersburg, MD, USA) as described previously.^56^ The qPCR primer sequences are listed in Table S7 (Supporting Information).

*Western Blotting*: Lysis, electrophoresis and target protein visualization were performed as described previously.^57^ Membranes were incubated overnight at 4 °C with primary anti‐YAP1, phos‐YAP1(ser127), CTGF, TEAD1, FSCN1, E‐cadherin, Vimentin, β‐catenin, CDK4, CyclinD1, p27, and GAPDH antibody (Cell Signaling Technology, Danvers, MA).

*Chromatin Immunoprecipitation (ChIP)*: ChIP assay was performed as described previously.^58^ Briefly, HCT116 cells were crosslinked in 1% formaldehyde for 10 min at 37 °C. DNA from fixed chromatin cells was then subjected to immunoprecipitation using a ChIP assay kit (Millipore, Billerica, MA, USA) and antibodies against TEAD1, YAP1, or antirabbit IgG (Santa Cruz Biotechnology, Santa Cruz, CA, USA) according to the manufacturer's protocol. The precipitated DNA fragments were purified and measured by qPCR under the conditions described above. Primers specific to each segment of interest are listed as follows: LINC00152‐pro (Site 1) Forward 5′‐GCACCTTTTCCCCACTTTCC‐3′ and Reverse: 5′‐GTGTCGCATTCAAAGATAATCCC‐3′; LINC00152‐pro (Site 2) Forward 5′‐GCTGTAGATGAAAACTGGGTCT‐3′ and Reverse: 5′‐ GCTTAGTACCTGATGCTGTGTAG‐3′; LINC00152‐pro (Site 3) Forward 5′‐AAGCATTTCCCCTGTGTGG‐3′ and Reverse: 5′‐GTCCCTGCAGCCCTTCCT‐3′.

*RNA‐Binding Protein Immunoprecipitation (RIP)*: RIP assay was performed as described previously,^59^ using the Magna RIP RNA Binding Protein Immunoprecipitation Kit (Millipore). Briefly, HCT116 cells were lysed by RIP lysis buffer. Then, 100‐µL cell extract was incubated with RIP buffer containing magnetic beads conjugated with human anti‐Ago2 antibody (Abcam, Cambridge, MA, USA) or negative control (normal mouse IgG, Millipore). After the antibody was recovered by protein A/G beads, RT‐qPCR was performed to detect *LINC00152* and microRNAs levels in the precipitates.

*RNA Pull‐Down Assay*: RNA pull‐down was performed as described previously.^60^ SW480 and HCT116 cells were transfected with biotinylated miRNA (40 nmol L^−1^) (Ribobio). After 48 h, the cells were harvested, and lysates were incubated with M‐280 streptavidin magnetic beads (Sigma–Aldrich, St. Louis, MO, USA). The bound RNAs were purified with Trizol reagent, and *LINC00152* mRNA levels were evaluated by qPCR.

*Cell Proliferation, Invasion, and Metastasis Assays*: Cell proliferation was assayed with CCK‐8 (Dojin Laboratories, Tokyo, Japan), tumor cell clone‐formation assay and flow cytometry analysis, which carried out as previously described.^61^ Cell migration and invasion were assays with wound healing assay and Matrigel invasion assay, which were performed as described previously.^62^

*Luciferase Assay*: Luciferase activity assays were performed with the Dual‐Luciferase Reporter Assay System (Promega, Madison, WI).

*Animal Study*: Animal care and euthanasia were approved by the Institutional Animal Care and Use Committee of Central South University (Changsha, China). Five‐week‐old male athymic BALB/c nude mice were randomly divided into four groups: 1) agomoir control, 2) LINC00152 overexpression + agomoir control, 3) LINC00152 overexpression + miR‐185‐3p agomir, and 4) LINC00152 overexpression + miR‐632 agomir, and were used for examining tumorigenicity. First, HCT116 cells (either stably overexpressing pEGFP‐LINC00152 or empty vector) were harvested after treatment of miR‐185‐3p agomir, miR‐632 agomir or control agomir (Ribobio) for 24 h, and single cell suspensions of 2 × 10^6^ cells were inoculated subcutaneously into the dorsal flanks of mice or injected into the tail vein of mice. Each group has eight nude mice. Seven days later, each group of mice were treated with miR‐185‐3p agomir, miR‐632 agomir, or control agomir (150 µL, 200 × 10^−9^ m) through injection, respectively. In the tumor growth xenograft model (Figure 6A), tumor volume was evaluated using the following formula: volume = (width + length)/2 × width × length × 0.5236; in the “tail vein–lung metastasis” nude mouse models (Figure 6F), the mice were killed after 60 days, and all the lungs are surgically removed. All tumor grafts were excised, weighed, harvested, fixed and embedded. Agomir is chemically modified double‐strand miRNA mimics which can mimic mature endogenous miRNAs after transfection into cells. The anti‐FSCN1 antibody (dilution 1:200, Cell Signaling Technology) and anti‐Ki67 antibody (dilution 1:100, Bioworld, Nanjing, China) were used to detect the protein expression levels of FSCN1 and the proliferation marker Ki67.

*In Situ Hybridization and Immunohistochemistry*: In situ hybridization was performed to detect *LINC00152* expression in tissue specimens. The *LINC00152* probes were 1) 5′‐CCTTC TTAGT CGTGT GTACA TCATT GGGAA TGGAG‐3′, 2) 5′‐CTCTA CCTGT TGCCC GCCGA TCACA GCCGG AATGC‐3′, and 3) 5′‐ATTTG CGGGT GGTCT GCCTG TGATA TTTTG GTCAT‐3′. The probes were synthetized and labeled with DIG‐dUTP at the 3′ end using a kit from Boster (# MK10098, Wuhan, China). In situ hybridization was performed as previously described.^63^

The protein expression of *FSCN1* was determined by Elivision two‐step immunohistochemical method in colorectal tissue microarray as described previously.^64^ In situ hybridization and immunohistochemical staining were independently evaluated at 200× magnification using light microscopy by two pathologists who were blinded to the clinicopathological data. A semiquantitative evaluation of *FSCN1* protein and *LINC00152* was performed using a method described in the previous work of the authors of this study.^65^

*Statistical Analysis*: All statistical analyses were carried out using SPSS version 20.0 and presented with Graphpad Prism Software 6.0. Data were expressed as mean ± s.e.m. Differences between two independent groups were tested with Student's *t*‐test. Correlations between different parameters were analyzed using a Spearman rank test. One‐way ANOVA followed by Tukey–Kramer multiple comparisons test was performed for comparing three or more groups within the same experiment. Survival curves were compared with the log‐rank (Mantel–Cox) test and the Gehan–Breslow–Wilcoxon test. *p*‐value of less than 0.05 was considered significant. Error bars represent standard deviations of three independent experimental measurements. All experiments were performed in triplicate, and repeated at least three times.

## Conflict of Interest

The authors declare no conflict of interest.

## Supporting information

Supporting Information
