# Supplementary material for: Targeting YAP1/LINC00152/FSCN1 Signaling Axis Prevents the Progression of Colorectal Cancer
Source: Adv Sci (Weinh). 2019 Dec 5;7(3):1901380. doi: 10.1002/advs.201901380 (PMC7001651; doi:10.1002/advs.201901380)
Supplement: Supplementary file 1 — Supporting Information [file ADVS-7-1901380-s001.pdf]

Copyright WILEY-VCH Verlag GmbH & Co. KGaA, 69469 Weinheim, Germany, 2019.

Supporting Information

for *Adv. Sci.,* DOI: 10.1002/advs.201901380

Targeting YAP1/LINC00152/FSCN1 Signaling Axis Prevents the Progression of Colorectal Cancer

Chunlin Ou, Zhenqiang Sun, Xiaoyun He, Xiaoling Li, Songqing Fan, Xiang Zheng, Qiu Peng, Guiyuan Li, Xiayu Li, and Jian Ma**

Targeting YAP1/LINC00152/FSCN1 signaling axis prevents the progression of colorectal cancer

Supporting Information

SI Methods and Materials

Data Mining and Analysis.

Six independent cohorts of primary colorectal cancer GSE data and their correlated clinic data, GSE14095, GSE20916, GSE9348, GSE23878, GSE41328 and GSE22598 were downloaded from the Gene Expression Omnibus (GEO) database. GSE14095 has 189 primary colorectal cancer samples; GSE20916 has 111 primary colorectal cancer samples; GSE9348 has 70 primary colorectal cancer samples and 12 normal colorectal samples; GSE23878 has 35 primary colorectal cancer samples and 24 normal colorectal samples; GSE41328 has 10 pairs of colorectal cancer and adjacent nontumor tissues; GSE22598 has 17 pairs of colorectal cancer and adjacent nontumor tissues, respectively.

Other CRC data were obtained from the TCGA database (http://cancergenome.nih.gov/). A total of 625 CRC tissues with gene expression profiles and 51 normal tissues which were non-tumor normal tissues from 51 CRC patients, were used to analyze the indicated genes expression levels between tumor and normal tissues.

Immunofluorescent Staining.

Cell lines were cultured on coverslips, rinsed with phosphate-buffered saline after 24 hours, and fixed with 4% paraformaldehyde for 20 min. Cells were permeabilized with 0.5% Triton X-100 for 30 min and blocked in phosphate-buffered saline (PBS) containing 5% goat serum for 90 min. Cells were incubated with primary monoclonal antibodies anti-E-cadherin, -Vimentin and -FSCN1 (Cell Signaling Technology) in blocking buffer at 4°C overnight. After washes in PBS, the coverslips were incubated for 1 h in the dark with secondary antibodies (Molecular Probes, Eugene, OR, USA). After further washing for three times, the slides were stained with DAPI for 5 min to visualize the nuclei, and viewed by confocal microscope (UltraView Vox; PerkinElmer, Waltham, MA).

Subcellular fractionation and RNA–FISH analysis.

To determine the cellular localization of LINC00152, cytoplasm and nuclear fractions were collected according to the manufacturer's instructions for the PARIS Kit (Life Technologies, USA).

Locked nucleic acid (LNA) modified probes for human LINC00152 and a negative/scramble control were used for RNA–FISH. The images were acquired using a confocal fluorescence microscope.

Supplementary Figures

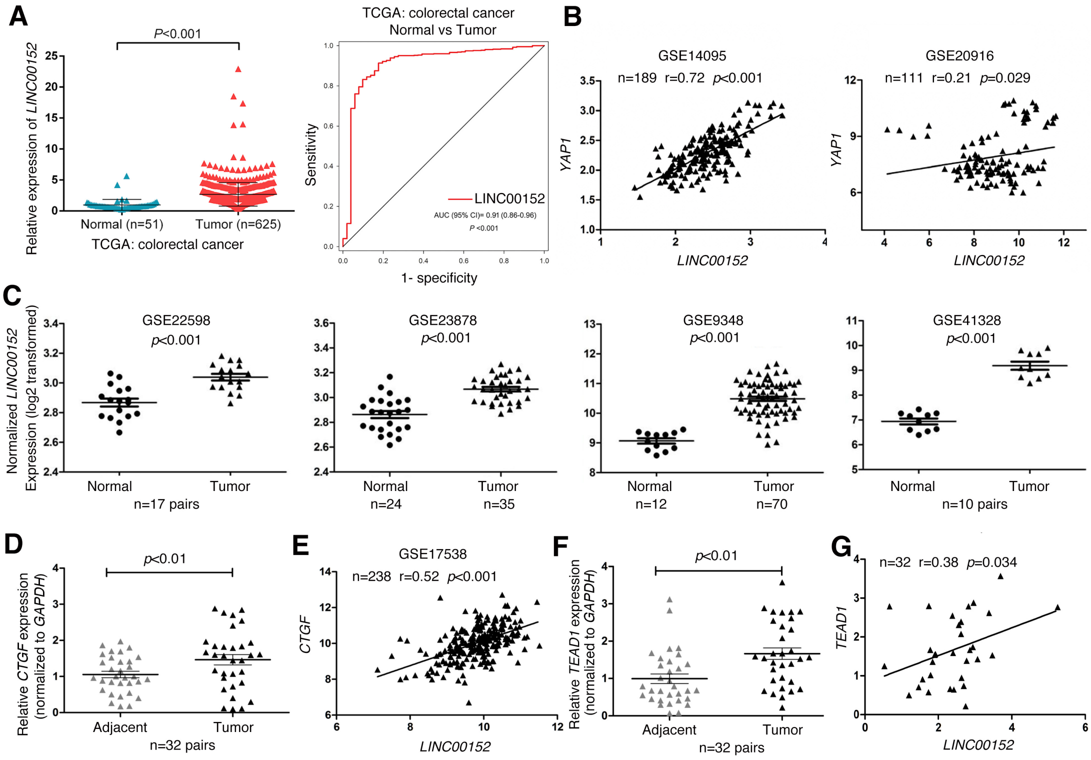

Supplementary Figure 1.

Bioinformatics analysis the *LINC00152* expression levels in CRC patients. **(A)** Left: *LINC00152* expression levels in TCGA CRC datasets; Right: ROC curves displaying the sensitivity and specificity of *LINC00152* expression in TCGA CRC datasets. **(B)** Correlation analysis between the expression levels of *YAP1* and *LINC0015* in GEO datasets (#GSE14095, GSE20916). **(C)** Relative expression of *LINC0015* in human normal colon tissues and cancer tissues were obtained from GEO database (#GSE22598, GSE23878, GSE9348 and GSE41328). (**D**) The expression of *CTGF* mRNA was analyzed by RT-qPCR in 32 pairs CRC samples and corresponding adjacent normal colorectal samples. (**E**) Correlation analysis between the expression levels of *LINC00152* and *CTGF* in the CEO datesets of GSE17538. (**F**) The expression of *TEAD1* mRNA was analyzed by RT-qPCR in 32 pairs CRC samples and corresponding adjacent normal colorectal samples. (**G**) Correlation analysis of *LINC00152* and *TEAD1* expression levels in 32 pairs CRC tissues by Spearman's rank correlation coefficient.

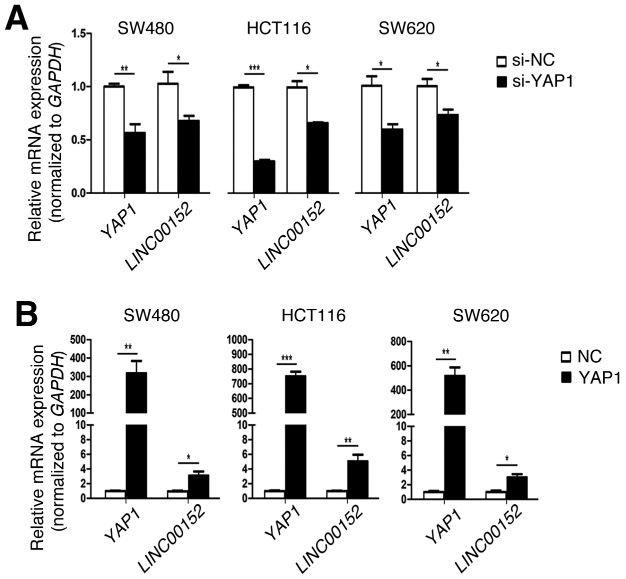

Supplementary Figure 2.

YAP1 regulates *LINC00152* expression. The expression level of *LINC00152* was detected in CRC cells transfected with indicated siRNAs (A) or plasmids (B). Data are shown as mean ± s.e.m. *p <0.05, **p <0.01, ***p <0.001 compared with control.

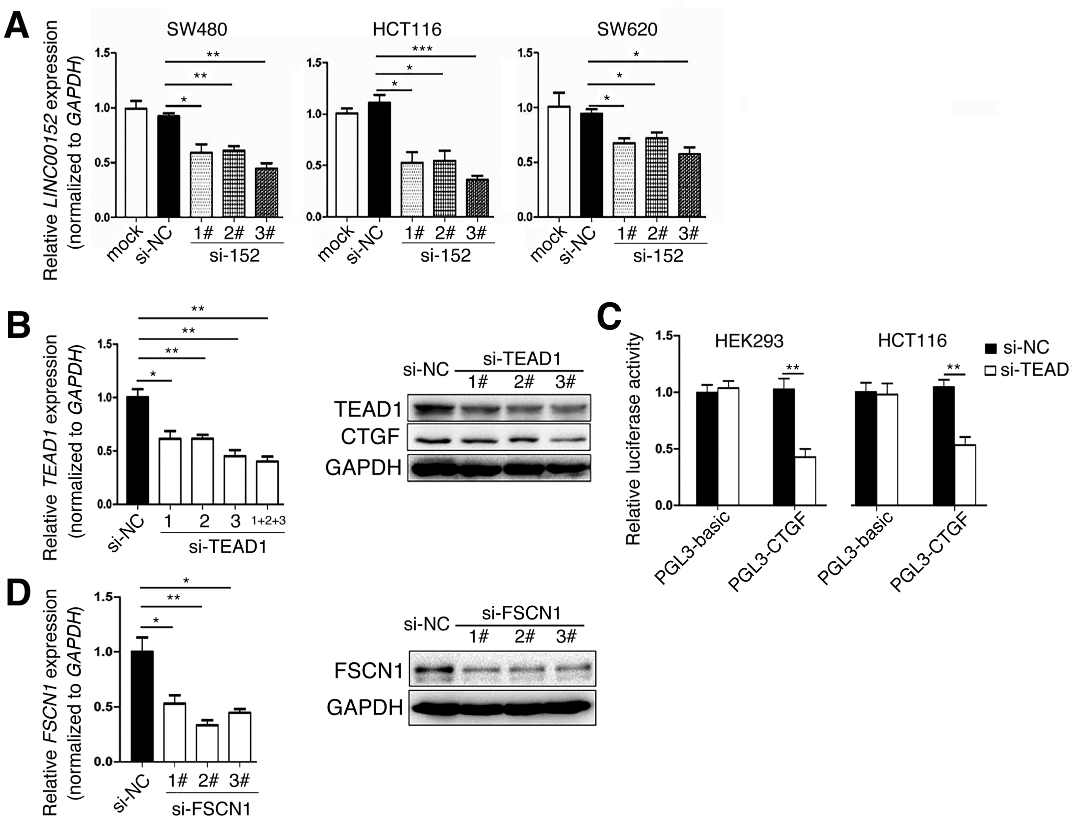

Supplementary Figure 3.

The interference efficiency of indicated siRNAs were verified in CRC cells. **(A)** After CRC cells transfected with either si-NC or si-*LINC00152* (1#, 2#, 3#) for 48 hrs, *LINC00152* levels were analyzed by RT-qPCR. **(B)** After cells transfected with either si-NC or si-*TEAD1*(1#, 2#, 3#) for 48 hrs, TEAD1 mRNA and protein levels were analyzed by RT-qPCR and Western blotting. (**C**) Cells were co-transfected with either si-NC or si-TEAD1 and a luciferase reporter containing the *CTGF* promoter (PGL3-CTGF) for 36 hrs. **(D)** After HCT116 cells transfected with either si-NC or si-*FSCN1*(1#, 2#, 3#) for 48 hrs, FSCN1 mRNA and protein levels were analyzed by RT-qPCR and Western blotting. Data are shown as mean ± s.e.m. *p <0.05, **p <0.01, ***p <0.001 compared with control.

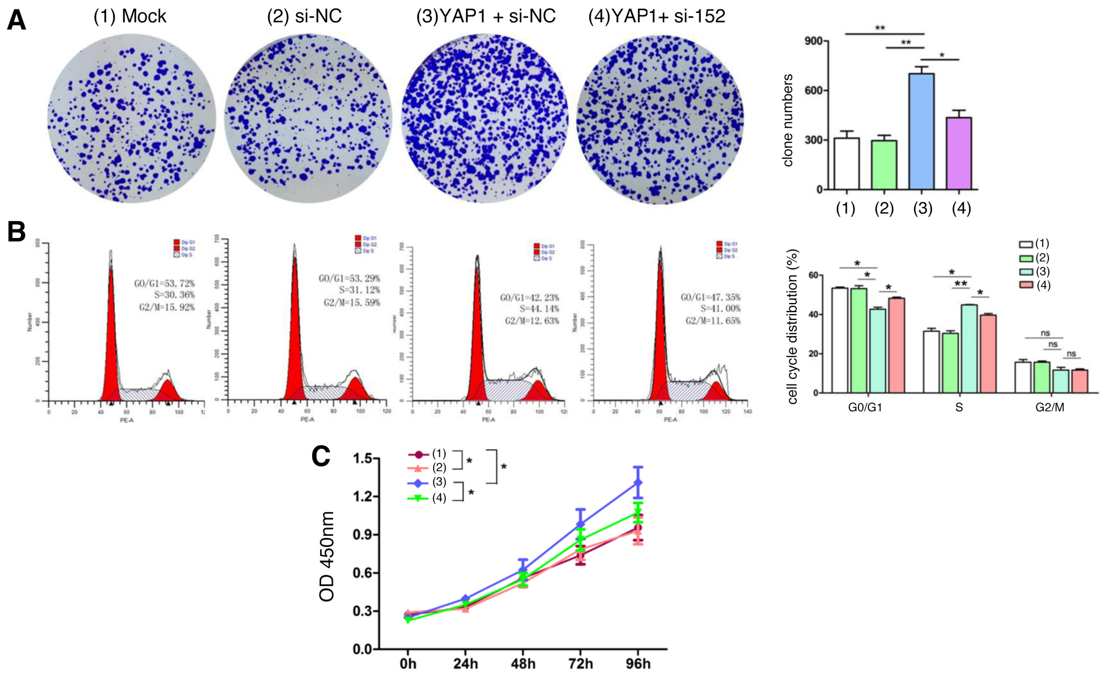

Supplementary Figure 4.

YAP1 promotes CRC cells tumorigenesis by regulating the *LINC00152* expression. HCT116 cells were transfected with indicated plasmids or siRNAs for 24 or 48 hrs. The cell proliferative ability was determined by cell clone-formation assay (**A**), cell-cycle analysis (**B**), and CCK8 assay (**C**). Data are shown as mean ± s.e.m. *p <0.05, **p <0.01 compared with control.

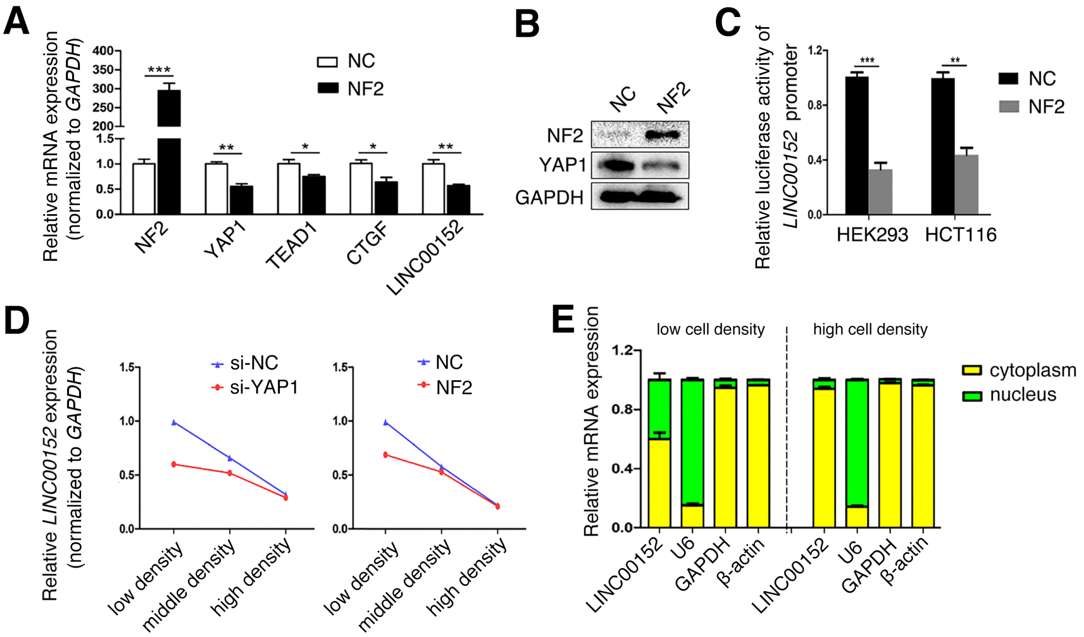

Supplementary Figure 5.

Cancer cell density regulates NF2/YAP1/LINC00152 axis. **(A)** Expression levels of *NF2*, *YAP1*, *TEAD1*, *CTGF* and *LINC00152* were detected by RT-qPCR in HCT116 cells transfected with NF2 overexpression plasmid or control plasmid. (**B**) Western blotting to measure NF2 and YAP1 protein levels in HCT116 cells transfected with NF2 overexpression plasmid or control plasmid for 48 hrs. (**C**) Cells were co-transfected with either NF2 overexpression plasmid or control plasmid and a luciferase reporter containing the *LINC00152* promoter for 36 hrs. (**D**) After HCT116 cells were transfected with indicated siRNAs or expression plasmids for 24 hrs, the cells were seeded at 1.0×10^3 (low cell density), 5.0×10^4 (medium cell density) and 8×10^5 (high cell density) cells per well (6-well plate). RT-qPCR assay was performed to measure the mRNA levels of *LINC00152*. (**E**) Nucleus and cytoplasm RNAs were analyzed for RT-qPCR to assay the expression levels of *LINC00152* in low and high cell density in HCT116 cells. U6 was used as nucleus RNA control, GPADH and β-Actin were used as cytoplasm RNA control. Data are shown as mean ± s.e.m. *p <0.05, **p <0.01, ***p <0.001 compared with control.

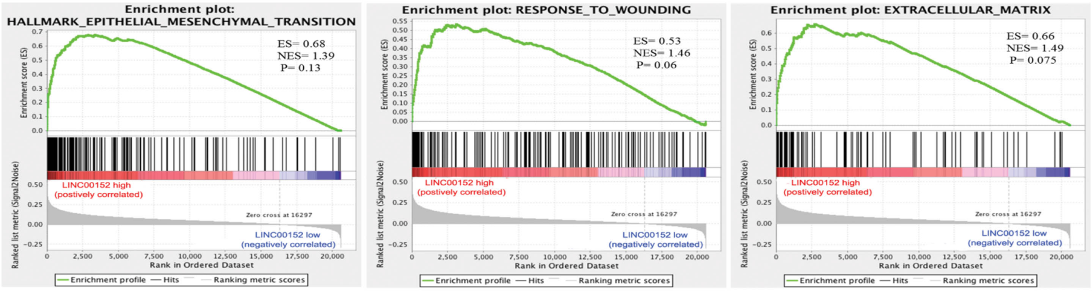

Supplementary Figure 6.

GSEA analysis showed the represented gene sets differences between *LINC00152*^{low} and *LINC00152*^{high} groups in GSE17538 dataset.

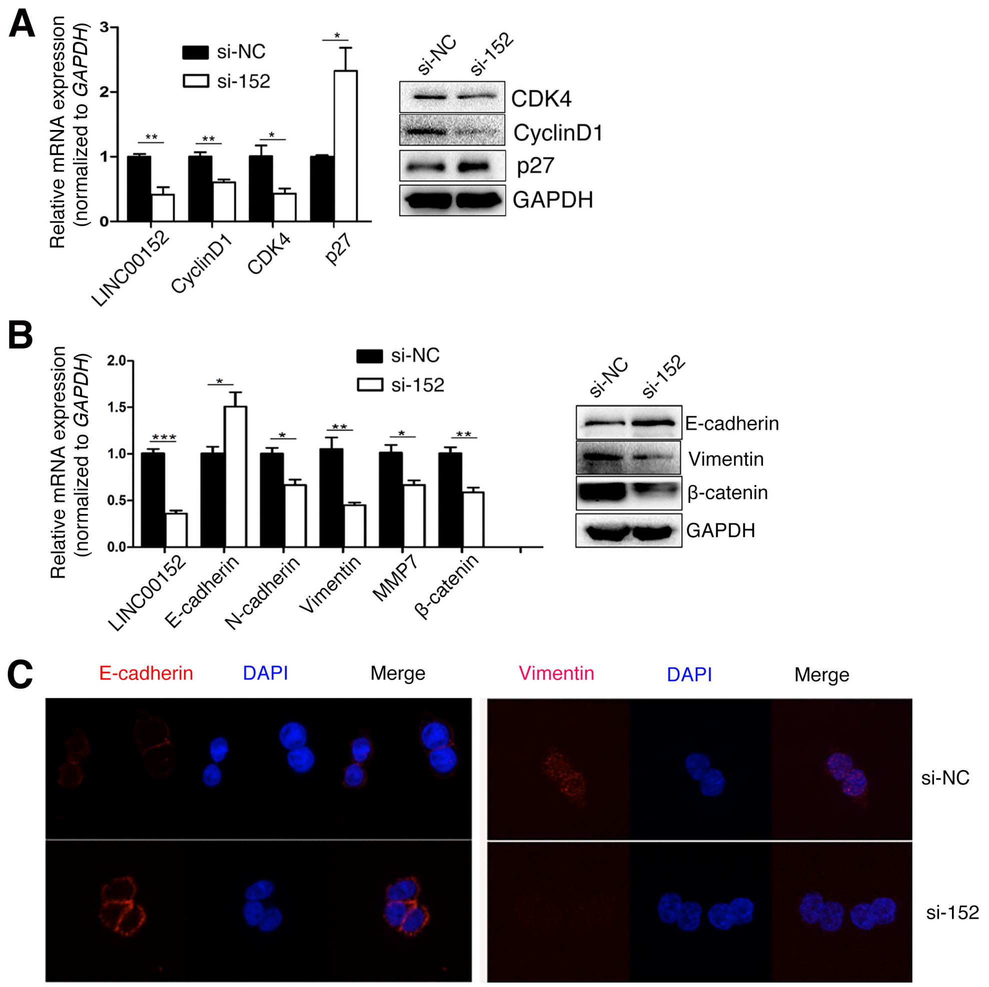

Supplementary Figure 7.

LINC00152 knockdown significantly suppressed the cell cycle and EMT-related molecules in CRC cells. HCT116 cells were transfected with indicated siRNAs for 48 hrs. (**A**) Expression levels of cell cycle-related molecules in HCT116 cells were assayed by RT-qPCR and Western blotting. (**B**) Expression levels of EMT-related molecules in HCT116 cells were assayed by RT-qPCR and Western blotting. (**C**) Confocal microscopy images of immunostaining for E-cadherin and Vimentin and merged with DAPI in HCT116 cells. Data are shown as mean ± s.e.m. *p <0.05, **p <0.01, ***p <0.001 compared with control.

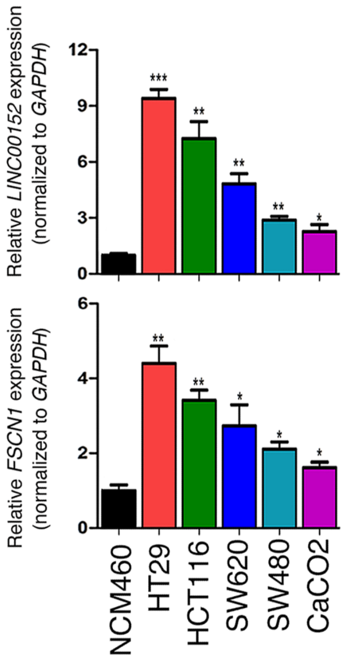

Supplementary Figure 8.

The mRNA expression patterns of *LINC00152* and *FSCN1* in normal colon mucosal cells (NCM460) and CRC cells (HT29, SW480, CaCO2, HCT116, SW620). Data are shown as mean ± s.e.m. *p <0.05, **p <0.01, ***p <0.001 compared with NCM460.

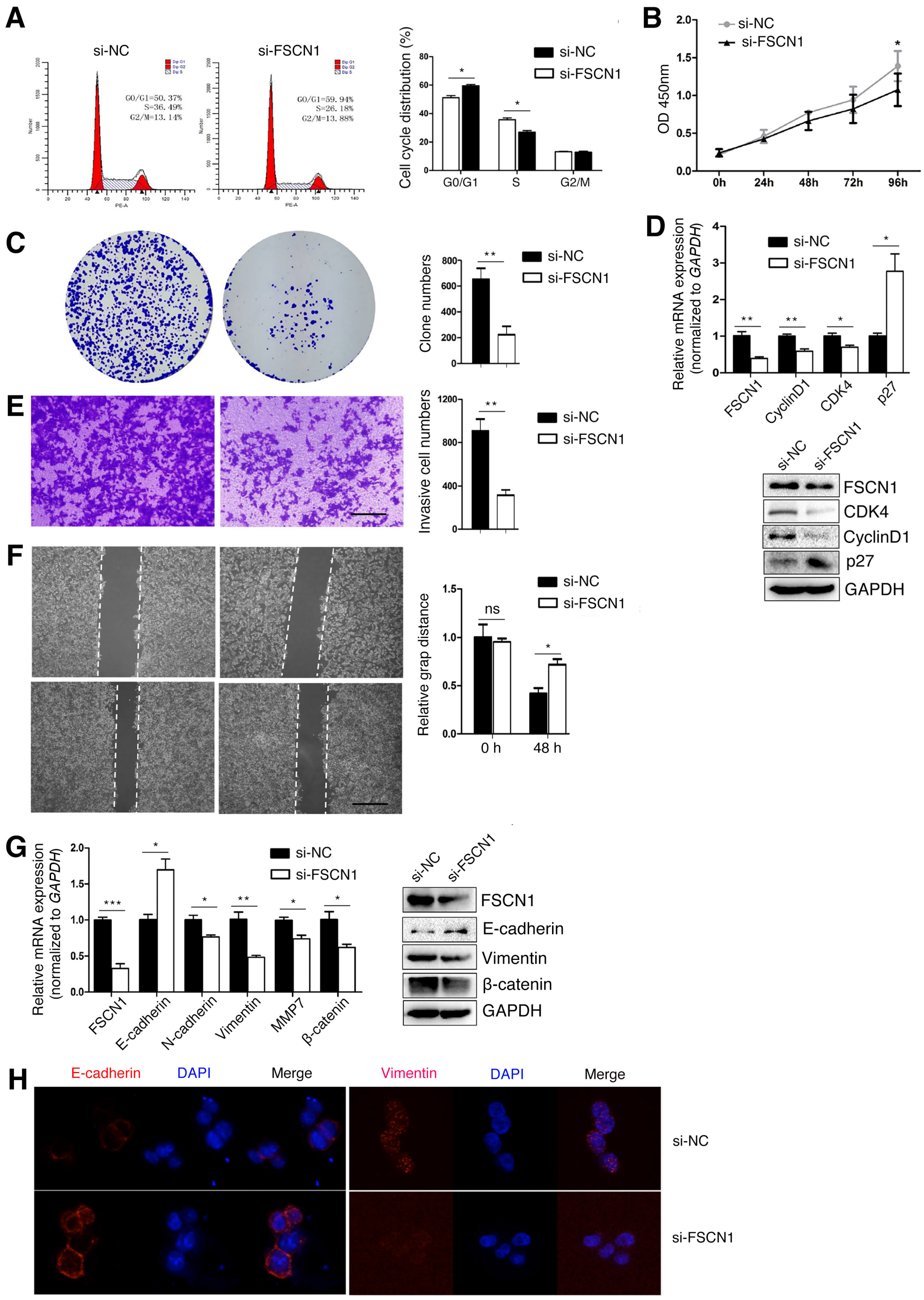

Supplementary Figure 9. *FSCN1* knockdown significantly suppressed the malignant phenotypes of CRC cells. HCT116 cells were transfected with indicated siRNAs for 24 or 48 hrs. The cell proliferative ability was determined by cell-cycle analysis (**A**), CCK8 assays (**B**) and cell clone-formation assay (**C**). (**D**) Expression levels of cell cycle-related genes in HCT116 cells were assayed by RT-qPCR and Western blotting. The cell invasive and migration abilities were determined by transwell matrigel assay (**E**) and wound-healing assay (**F**). (**G**) Expression levels of EMT-related genes in HCT116 cells were assayed by RT-qPCR and Western blotting. (**H**) Confocal microscopy images of immunostaining for E-cadherin and Vimentin and merged with DAPI in HCT116 cells transfected with si-NC or si-FSCN1. Data are shown as mean ± s.e.m. *p <0.05, **p <0.01, ***p <0.001 compared with control.

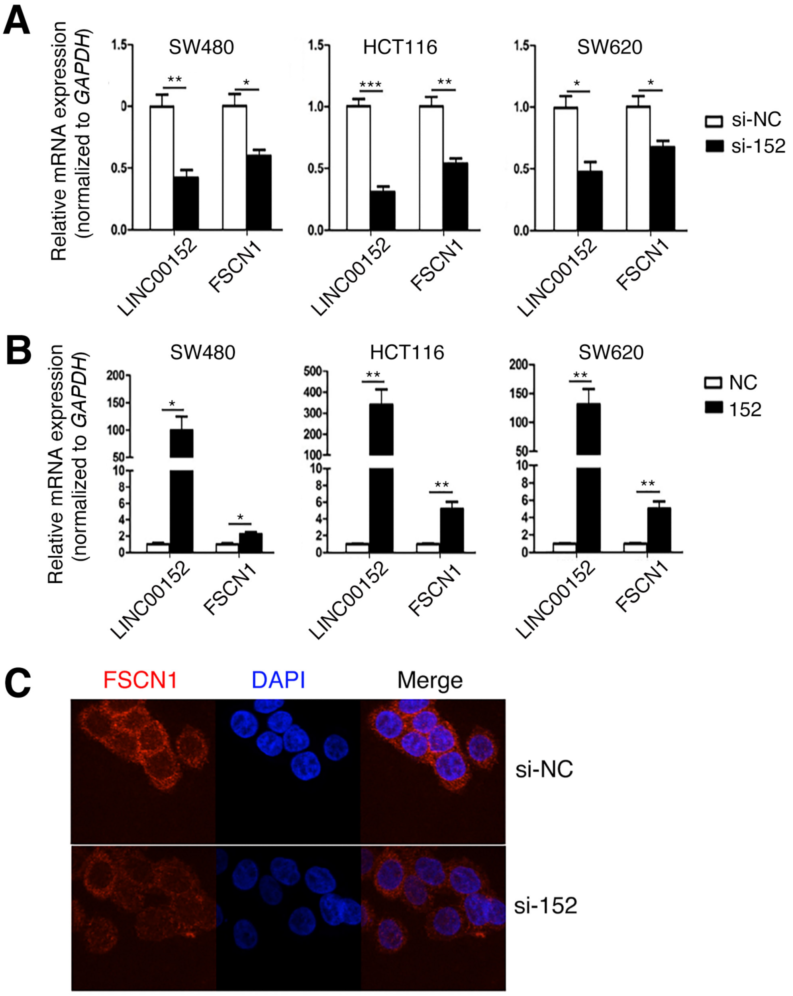

Supplementary Figure 10.

LINC00152 regulated the FSCN1 expression in CRC cells. (**A-B**) The mRNA expression level of *FSCN1* was detected in CRC cells transfected with indicated siRNAs (A) or plasmids (B). (**C**) Confocal microscopy images of immunostaining for FSCN1 protein and merged with DAPI in HCT116 cells transfected with si-NC or si-152. Data are shown as mean ± s.e.m. *p <0.05, **p <0.01, ***p <0.001 compared with control.

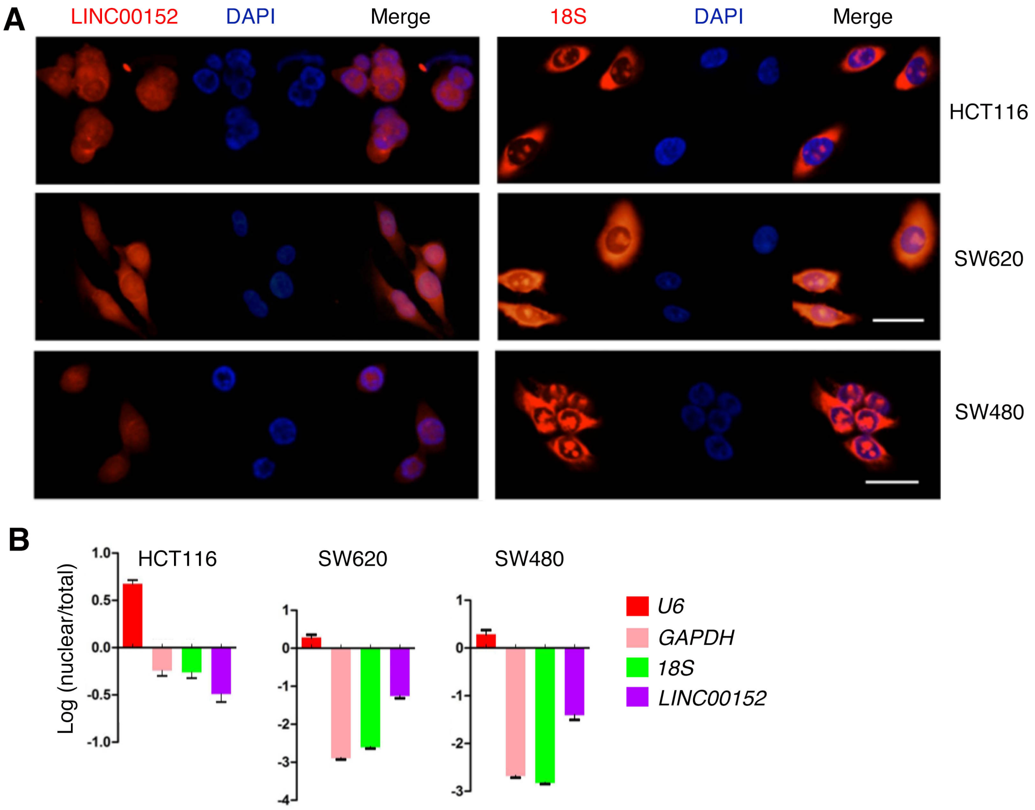

Supplementary Figure 11.

Analysis of the subcellular localization of LINC00152 in CRC cells were measured by RNA-FISH assay (**A**) and cytoplasm/nucleus isolation assays (**B**). U6 was used as nucleus RNA control, GPADH and β-Actin were used as cytoplasm RNA control.

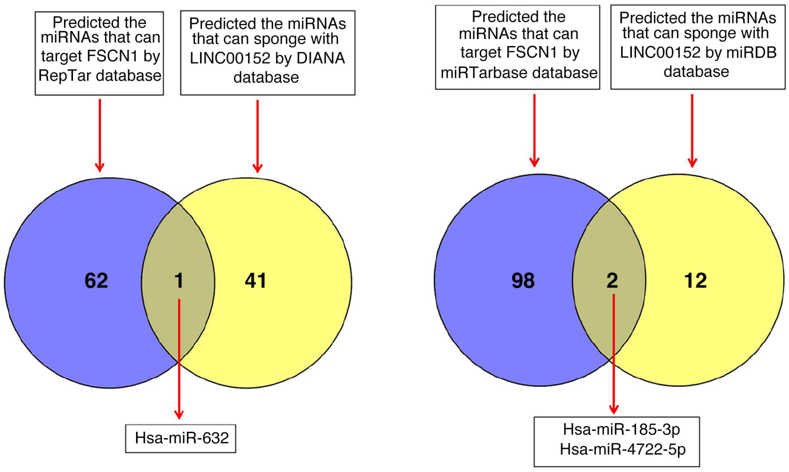

Supplementary Figure 12.

Bioinformatic prediction of the potential microRNAs that have binding sites both with LINC00152 and 3'-UTR of *FSCN1*.

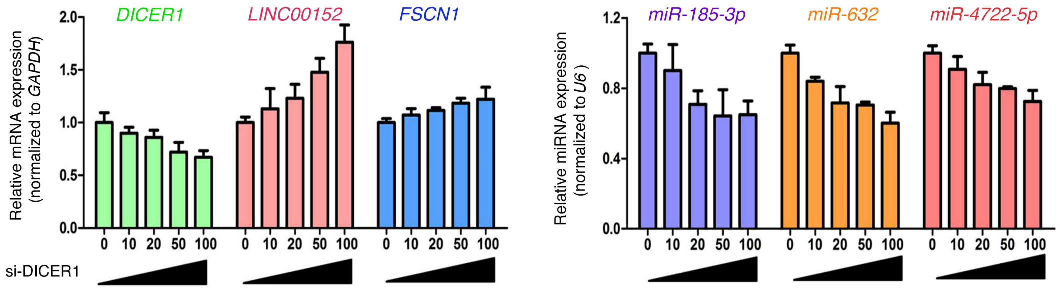

Supplementary Figure 13.
HCT116 cells were transfected with different amounts of si-DICER1 for 48 hrs, and the indicated genes and microRNAs' expression levels were assayed by RT-qPCR. Data are shown as mean ± s.e.m.

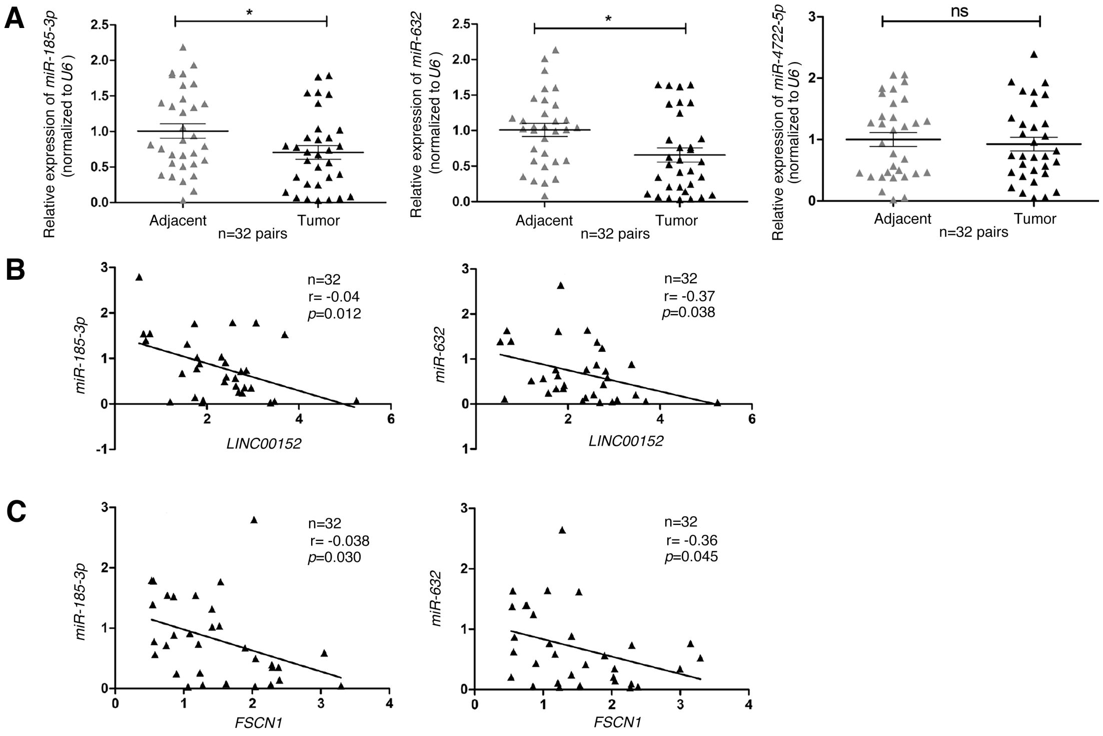

Supplementary Figure 14.

Clinical data of miR-185-3p, miR-632 and miR-4722-5p from bioinformatics analysis. (**A**) The expression of miR-185-3p, miR-632 and miR-4722-5p were analyzed by RT-qPCR in 32 pairs CRC samples and corresponding adjacent normal colorectal samples. (**B**) Correlation analysis between the expression levels of miR-185-3p or miR-632 and LINC00152 in 32 pairs CRC tissues by Spearman's rank correlation coefficient. (**C**) Correlation analysis between the expression levels of miR-185-3p or miR-632 and FSCN1 in 32 pairs CRC tissues by Spearman's rank correlation coefficient. *p <0.05 compared with control.

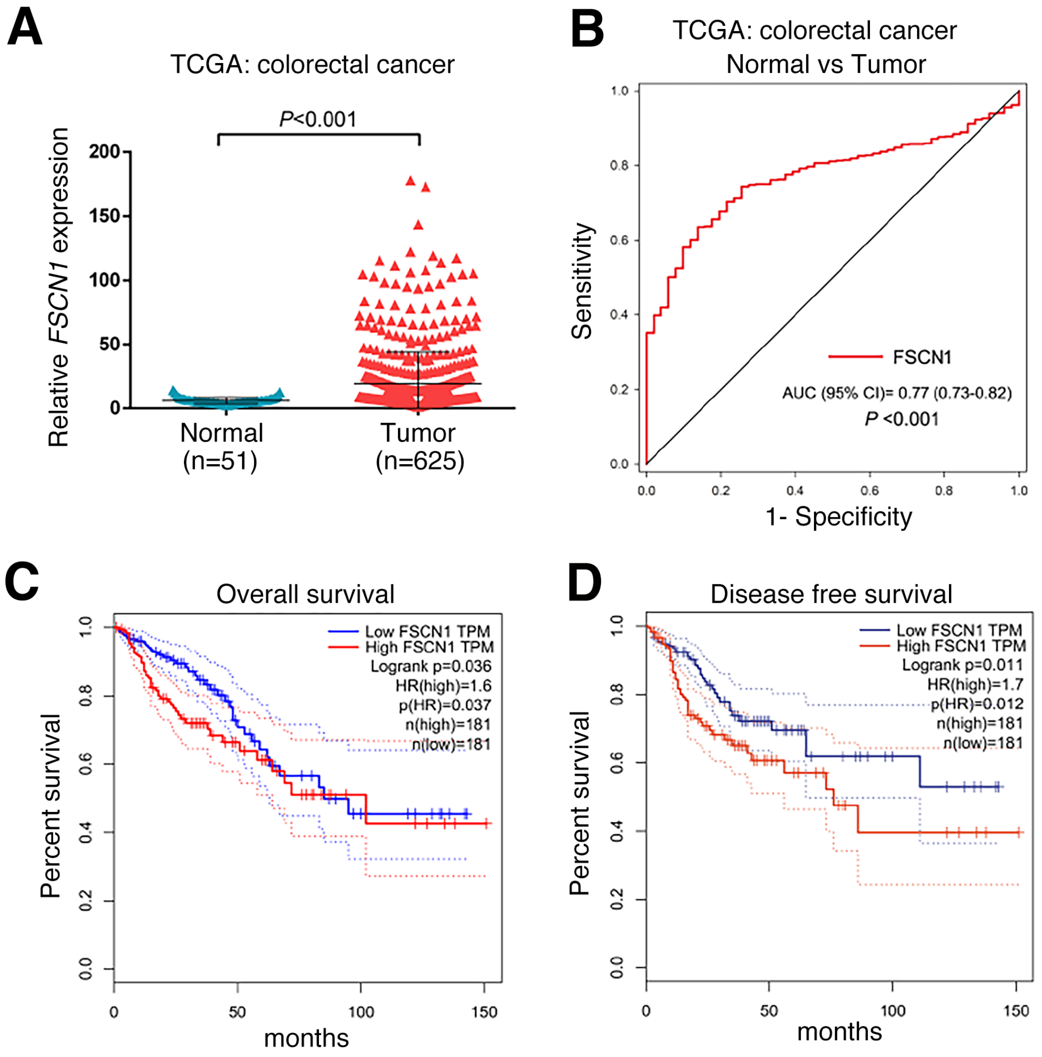

Supplementary Figure 15.

Clinical data of FSCN1 from bioinformatics analysis. **(A-B)** Analysis of *FSCN1* expression in the CRC dates of TCGA; Receiver-operating characteristic (ROC) curves displaying the sensitivity and specificity of *FSCN1* in the CRC dates of TCGA. **(C-D)** GEPIA database was used to analyze the clinical impact of *FSCN1* expression patterns on the CRC patients' overall survival (OS) and disease-free survival (DFS).

Supplementary Tables

Supplementary Table 1.
The differentially expressed lncRNAs induced by si-*YAP1* in HCT116 cells

The downregulated lncRNAs induced by si-*YAP1*:

Gene symbol	Probe set ID	Fold change	*P* value
AC017002.2	1562056_at	-1.977299	0.000155
LINC00152	225799_at	-1.785264	0.000171
LOC102723721	235921_at	-1.58727	0.000186
FLJ39051	227925_at	-1.474931	0.000601
LOC387723	229323_at	-1.45104	0.002543
C14orf139	219563_at	-1.423399	0.005027
NCRNA00188	239754_at	-1.397291	0.00054
MALAT1	224559_at	-1.389849	0.00076
TUG1	212337_at	-1.382821	0.003185
LOC100131138	230195_at	-1.362809	0.001934
LOC92249	212957_s_at	-1.305471	0.001775
FLJ42709	1556695_a_at	-1.305122	0.005343
NCRNA00202	231196_x_at	-1.290118	0.005704
LOC148189	235191_at	-1.281432	0.003895
LOC643837	1557055_s_at	-1.279309	0.006953
MALAT1	228582_x_at	-1.264915	0.005347
TTC28-AS	244189_at	-1.249074	0.005909
C1orf97	224444_s_at	-1.246075	0.003642
LOC389906	1569629_x_at	-1.245446	0.004817
UCA1	227919_at	-1.235564	0.00754

The upregulated lncRNAs induced by si-*YAP1*:

Gene symbol	Probe set ID	Fold change	*P* value
LOC101929752	1561060_at	1.510043	0.000339
BC037833	1569765_at	1.550434	0.000592
LOC100128822	235174_s_at	1.940818	0.00044

Supplementary Table 2.

The dysregulated lncRNAs mined from the GSE41328 data set (analyzed by SAM)

Gene ID	lncRNA Name	Score(d)	Numerator(r)	Fold Change	q-value(%)
225799_at	LINC00152	1.890	50	4.901	0.000
225857_s_at	LOC388796	1.890	50	2.526	0.000
224915_x_at	NCRNA00275	1.890	50	2.608	0.000
226227_x_at	NCRNA00275	1.890	50	2.603	0.000
225699_at	C7orf40	1.890	50	2.395	0.000
224741_x_at	GAS5	1.852	49	2.285	0.000
224841_x_at	GAS5	1.852	49	2.336	0.000
1558290_a_at	PVT1	1.587	42	2.009	0.000
221621_at	C17orf86	1.474	39	1.516	0.000
227517_s_at	GAS5	1.474	39	1.945	0.000
224646_x_at	H19	1.285	34	15.142	0.000
1560089_at	LOC100289019	-1.890	-50	0.499	0.000
1560010_a_at	FLJ32063	-1.890	-50	0.412	0.000
229385_s_at	PLAC2	-1.890	-50	0.413	0.000
236001_at	LOC400573	-1.890	-50	0.353	0.000
241418_at	LOC344887	-1.890	-50	0.584	0.000
222307_at	LOC282997	-1.890	-50	0.589	0.000
1553420_at	FLJ32063	-1.814	-48	0.462	0.000
214696_at	C17orf91	-1.814	-48	0.554	0.000
237450_at	LOC389332	-1.814	-48	0.564	0.000
207259_at	C17orf73	-1.776	-47	0.563	0.000
236351_at	LOC389023	-1.739	-46	0.206	0.000
228601_at	LOC401022	-1.739	-46	0.387	0.000
227969_at	LOC400960	-1.739	-46	0.544	0.000
240838_s_at	LOC145837	-1.701	-45	0.507	0.000
1559884_at	CDKN2B-AS	-1.625	-43	0.632	0.000
1557548_at	C10orf108	-1.625	-43	0.643	0.000
230245_s_at	LOC283663	-1.550	-41	0.585	0.000
242649_x_at	C15orf21	-1.550	-41	0.562	0.000
224870_at	KIAA0114	-1.247	-33	0.618	0.000

NOTE: The red marks represent the upregulated genes in tumor comparing to normal tissue (Fold Change>1); the green marks represent the downregulated genes in tumor comparing to normal tissue (Fold Change<1).

Supplementary Table 3.

The dysregulated lncRNAs mined from the GSE9348 data set (analyzed by SAM)

Gene ID	lncRNA Name	Score(d)	Numerator(r)	Fold Change	q-value(%)
225799_at	LINC00152	1.883	287	1.610	0.000
225857_s_at	LOC388796	2.565	391	3.521	0.000
224915_x_at	NCRNA00275	2.689	410	3.022	0.000
226227_x_at	NCRNA00275	2.611	398	2.617	0.000
225699_at	C7orf40	2.716	414	3.075	0.000
224741_x_at	GAS5	1.856	283	1.864	0.000
224841_x_at	GAS5	1.942	296	1.891	0.000
1558290_a_at	PVT1	2.722	415	5.086	0.000
221621_at	C17orf86	1.883	287	1.858	0.000
227517_s_at	GAS5	2.565	391	2.328	0.000
224646_x_at	H19	2.742	418	5.140	0.000
1560089_at	LOC100289019	-2.696	-411	0.374	0.000
1560010_a_at	FLJ32063	-2.184	-333	0.380	0.000
229385_s_at	PLAC2	-2.755	-420	0.123	0.000
236001_at	LOC400573	-2.755	-420	0.140	0.000
241418_at	LOC344887	-2.670	-407	0.252	0.000
222307_at	LOC282997	-2.289	-349	0.409	0.000
1553420_at	FLJ32063	-2.250	-343	0.374	0.000
214696_at	C17orf91	-2.755	-420	0.220	0.000
237450_at	LOC389332	-2.381	-363	0.369	0.000
207259_at	C17orf73	-2.735	-417	0.219	0.000
236351_at	LOC389023	-2.434	-371	0.371	0.000
228601_at	LOC401022	-2.755	-420	0.054	0.000
227969_at	LOC400960	-2.722	-415	0.308	0.000
240838_s_at	LOC145837	-2.611	-398	0.092	0.000
1559884_at	CDKN2B-AS	-2.598	-396	0.083	0.000
1557548_at	C10orf108	-2.152	-328	0.479	0.000
230245_s_at	LOC283663	-1.955	-298	0.584	0.000
242649_x_at	C15orf21	-2.755	-420	0.178	0.000
224870_at	KIAA0114	-1.856	-283	0.556	0.000

NOTE: The red marks represent the upregulated genes in tumor comparing to normal tissue (Fold Change>1); the green marks represent the downregulated genes in tumor comparing to normal tissue (Fold Change<1).

Supplementary Table 4.

The top 10 differentially expressed genes induced by si-*LINC00152* in HCT116 cells

The top 10 upregulated genes induced by si-*LINC00152*:

Gene name	Gene description	Fold change	P value
C9orf66	chromosome 9 open reading frame 66	4.036310537	0.034307306
ZNF780A	zinc finger protein 780A	2.833034853	0.004767065
ATF6B	activating transcription factor 6 beta	2.717506402	8.96E-10
TCEA3	transcription elongation factor A (SII), 3	2.602518399	0.000347284
ZNF251	zinc finger protein 251	1.72483619	0.009401668
HNRNPL	heterogeneous nuclear ribonucleoprotein L	1.643896359	0.001158746
SNAI2	snail family zinc finger 2	1.585791269	0.049023102
SLC26A7	solute carrier family 26 (anion exchanger), member 7	1.532517494	0.011510112
RASA3	RAS p21 protein activator 3	1.4508939	0.000255514
IGSF1	immunoglobulin superfamily, member 1	1.33555582	0.028843266

The top 10 downregulated genes induced by si-*LINC00152*:

Gene name	Gene description	Fold change	P value
PSMC4	proteasome (prosome, macropain) 26S subunit, ATPase, 4	-3.530110247	0.002348774
SCNN1B	sodium channel, non voltage gated 1 beta subunit	-3.033870649	0.018140184
CCBE1	collagen and calcium binding EGF domains 1	-2.93298222	0.006545299
ACSM1	acyl-CoA synthetase medium-chain family member 1	-2.421584107	0.033480132
PRUNE2	prune homolog 2 (Drosophila)	-2.40252383	0.049244241
SPRR2E	small proline-rich protein 2E	-2.339003156	0.039539877
LCK	LCK proto-oncogene, Src family tyrosine kinase	-2.324922973	0.013345354
CCDC63	coiled-coil domain containing 63	-2.20637796	0.03446566
SH2D1B	SH2 domain containing 1B	-2.097279155	0.033164655
ORM2	orosomucoid 2	-2.066132519	0.047997489
WNT6	wingless-type MMTV integration site family, member 6	-1.968790039	0.001657408

Supplementary Table 5.

The top 20 differentially expressed pathways induced by si-*LINC00152* in HCT116 cells

The top 20 upregulated pathways induced by si-*LINC00152*:

Pathway	P value	Enrichment score
TNF signaling pathway	4.32E-05	9.839160839
Amphetamine addiction	9.60E-05	12.11538462
Pertussis	0.000149138	10.82307692
Galactose metabolism	0.000170319	18.03846154
Estrogen signaling pathway	0.00043491	8.199300699
Cholinergic synapse	0.000671409	7.312889813
Cocaine addiction	0.000737722	11.04395604
N-Glycan biosynthesis	0.000737722	11.04395604
Glycosaminoglycan biosynthesis - keratan sulfate	0.001339033	18.03846154
Colorectal cancer	0.001466909	8.728287841
Apoptosis	0.001597962	5.798076923
Hepatitis B	0.001865116	5.559799789
HTLV-I infection	0.002229005	4.194991055
B cell receptor signaling pathway	0.002348293	7.413066386
Leishmaniasis	0.002348293	7.413066386
RNA degradation	0.002734983	7.027972028
Salmonella infection	0.00374307	6.292486583
Transcriptional misregulation in cancers	0.003988044	4.509615385
Glycosphingolipid biosynthesis - lacto and neolacto series	0.004042408	10.40680473
Rheumatoid arthritis	0.004255008	6.012820513

The top 20 downregulated pathways induced by si-*LINC00152*:

Pathway	P value	Enrichment score
Inflammatory mediator regulation of TRP channels	0.000623958	4.722744361
Steroid biosynthesis	0.001210289	9.256578947
Butirosin and neomycin biosynthesis	0.002335425	13.22368421
Butanoate metabolism	0.003618039	6.38384755
Tight junction	0.003756947	3.329704657
Circadian rhythm	0.0043825	5.971986418
Propanoate metabolism	0.00479866	5.785361842
Sulfur relay system	0.004900394	9.256578947
Synthesis and degradation of ketone bodies	0.007087434	7.713815789
Biosynthesis of amino acids	0.008179324	3.752667141
Fatty acid biosynthesis	0.008317765	7.120445344
Aldosterone-regulated sodium	0.008377255	4.746963563

reabsorption		
Leukocyte transendothelial migration	0.008740427	3.137823372
cGMP - PKG signaling pathway	0.009081605	2.771430823
Pyruvate metabolism	0.009622287	4.515404365
Folate biosynthesis	0.009636577	6.611842105
Platelet activation	0.010016971	3.034943917
TGF-beta signaling pathway	0.012652824	3.305921053
2-Oxocarboxylic acid metabolism	0.014104026	5.44504644
Nitrogen metabolism	0.014104026	5.44504644

Supplementary Table 6.
The siRNA sequences of targeting genes

Targeting Gene	Nos.	Sequence
si-h-YAP1-1	siG000010413A	GCGTAGCCAGTTACCAACA
si-h-YAP1-2	siG000010413B	CAGTGGCACCTATCACTCT
si-h-YAP1-3	siG000010413C	GGTGATACTATCAACCAAA
si-h-LINC00152-1	siB150910084421	GGGAAATAAATGACTGGAT
si-h-LINC00152-2	siB150910084447	GGAGATGAAACAGGAAGCT
si-h-DICER1-1	siB0965140932	GAATCAGCCTCGCAACAAA
si-h-DICER1-2	siB0862949570	TGCTTGAAGCAGCTCTGGA
si-h-DICER1-3	siB08106144559	CCTCCAGCAGTCCCTAGGA
si-h-FSCN1-1	siB0837153452	CAAAGACTCCACAGGCAAA
si-h-FSCN 1-2	siB0837153513	GCTGCTACTTTGACATCGA
si-h-FSCN 1-3	siB08137153533	GCAAGTTTGTGACCTCCAA
si-h-TEAD1-1	siG000007003A	GGATCCTCACAAGACGTCA
si-h-TEAD 1-2	siG000007003B	GCTCCATTGGCACAACCAA
si-h-TEAD 1-3	siG000007003C	GTAACCAGTCAGTACGAGA
si-h-FAT4	siG160428071011	GACGATTAGTGCTATAGAT
si-h-LATS1	siG160428071011	ACUUUGCCGAGGACCCGAA
si-h-LATS2	siG160428071011	GUUCGGACCUUAUCAGAAA
si-h-MST1	siG160428071011	CAAGCGAAAUACAGUGAUATT
si-h-MST2	siG160428071011	GGAUAGUUUUUCAAAUAGG
si-h-SAV1	siB170817011724	AAAUUCGGAUGACUCAACUCGUUCC
si-h-CD44	siG160428071011	CGUGGAGAAAAAUGGUCGC
si-h-YAP1-1	siG000010413A	GCGTAGCCAGTTACCAACA
si-h-YAP1-2	siG000010413B	CAGTGGCACCTATCACTCT

Supplementary Table 7.

Primer sequences for real-Time PCR

**Gene (human)**	**Primer (Forward)**	**Primer (Reverse)**
YAP1	TAGCCCTGCGTAGCCAGTTA	TCATGCTTAGTCCACTGTCTGT
LINC00152	TGGGAATGGAGGGAAATAAA	CCAGGAACTGTGCTGTGAAG
TEAD1	GGCTTCGGCTTGGAAAATCC	ACTAGACACCTGTTTTCTGGTCC
CTGF	GGCCTCTTCTGCGATTTCG	GCAGCTTGACCCTTCTCGG
FSCN1	CACACGGGCAAGTACTGGAC	TCTGAGTCCCCTGCTGTCTC
DICER1	CCCGTTCCCCTGTGCGAGAATT	TTCGGAGGGCTCTTCTTGCTGC
E-Cadherin	TGAAGCCCCCATCTTTGTGC	GGCTGTGTACGTGCTGTTCT
N-Cadherin	ATCCTACTGGACGGTTCG	TTGGCTAATGGCACTTGA
ZEB1	GCACAACCAAGTGCAGAAGA	GCCTGGTTCAGGAGAAGATG
ZEB2	GATGAAATAAGGGAGGGTGG	CCTCAAAATCTGATGTGCAA
Vimentin	CGGGAGAAATTGCAGGAGGAGA	TCTTGGCAGCCACACTTTCAT
MMP7	GGCTTTAAACATGTGGGGCA	GACTGCTACCATCCGTCCAG
MMP11	TCATGATCGACTTCGCCAGG	CAGTGGGTAGCGAAAGGTGT
MMP13	GCCATTACCAGTCTCCGAGG	TACGGTTGGGAAGTTCTGGC
β-Catenin	GGCGCCATTTTAAGCCTCTC	CAAATACCCTCAGGGGAACAG
CyclinD1	GCTGCGAAGTGGAAACCATC	CCTCCTTCTGCACACATTTGAA
CyclinE1	GGCCAAAATCGACAGGACGG	ATCCGAGGCTTGCACGTTGAGT
CDK2	AGCTGTGGACATCTGGAGCCTG	CCCAACCTTGTGATGCAGCCA
CDK4	TTGGTGTCGGTGCCTATGGG	CCATCAGCCGGACAACATTGGG
CCNA2	CTGCATCTCTGGGCGTCTTT	GTGCAACCCGTCTCGTCTTC
β-Actin	TCACCAACTGGGACGACATG	GTCACCGGAGTCCATCACGAT
18S	GTGGGCCGAAGATATGCTCA	TTGGCTAGGACCTGGCTGTA
U6	CTCGCTTCGGCAGCACA	AACGCTTCACGAATTTGCGT
GAPDH	AACGGATTTGGTCGTATTGG	TTGATTTTGGAGGGATCTCG
